# The patterns of deleterious mutations during the domestication of soybean

**DOI:** 10.1038/s41467-020-20337-3

**Published:** 2021-01-04

**Authors:** Myung-Shin Kim, Roberto Lozano, Ji Hong Kim, Dong Nyuk Bae, Sang-Tae Kim, Jung-Ho Park, Man Soo Choi, Jaehyun Kim, Hyun-Choong Ok, Soo-Kwon Park, Michael A. Gore, Jung-Kyung Moon, Soon-Chun Jeong

**Affiliations:** 1 Bio-Evaluation Center, Korea Research Institute of Bioscience and Biotechnology, Cheongju, Chungbuk 28116 Korea; 2 Plant Immunity Research Center, Plant Genomics and Breeding Institute, College of Agriculture and Life Sciences, Seoul National University, Seoul, 08826 Korea; 3 Plant Breeding and Genetics Section, School of Integrative Plant Science, Cornell University, Ithaca, NY 14853 USA; 4 Department of Life Science, The Catholic University of Korea, Bucheon, 14662 Korea; 5 National Institute of Crop Science, Rural Development Administration, Wanju, Jeonbuk 55365 Korea; 6 Agricultural Genome Center, National Academy of Agricultural Sciences, Rural Development Administration, Jeonju, Jeonbuk 55365 Korea

**Keywords:** Genetic variation, Agricultural genetics

## Abstract

Globally, soybean is a major protein and oil crop. Enhancing our understanding of the soybean domestication and improvement process helps boost genomics-assisted breeding efforts. Here we present a genome-wide variation map of 10.6 million single-nucleotide polymorphisms and 1.4 million indels for 781 soybean individuals which includes 418 domesticated (*Glycine max*), 345 wild (*Glycine soja*), and 18 natural hybrid (*G. max*/*G. soja*) accessions. We describe the enhanced detection of 183 domestication-selective sweeps and the patterns of putative deleterious mutations during domestication and improvement. This predominantly selfing species shows 7.1% reduction of overall deleterious mutations in domesticated soybean relative to wild soybean and a further 1.4% reduction from landrace to improved accessions. The detected domestication-selective sweeps also show reduced levels of deleterious alleles. Importantly, genotype imputation with this resource increases the mapping resolution of genome-wide association studies for seed protein and oil traits in a soybean diversity panel.

## Abstract (web summary)

The accumulation of recombination events in selfing species may lead to a rapid fixation of both beneficial and deleterious mutations. Here, the authors resequence 781 soybean accessions, show purging of deleterious mutation during domestication, and report genome-wide associations for seed protein and oil traits.

## Introduction

Soybean (*Glycine max* [L.] Merr.) is a globally important crop species, as it is a major source of seed protein and oil. Cultivated soybean (*G. max*) was domesticated an estimated 7000–9000 years ago from wild soybean (*Glycine soja* Sieb. & Zucc.) with distribution in East Asia^1,2^. The cultivation of soybean has been historically confined to East Asia and only recently expanded to North America, South America, and India, positioning it as one of the top crops in terms of growing area worldwide^3^. Both wild and domesticated soybean are predominantly selfing^4^. The accumulation of recombination events across generations in such selfing plant species may result in rapid fixation and weak selection of both favorable and deleterious mutations^5^. Furthermore, the reduced effective population size due to a genetic bottleneck during domestication will enhance the stochasticity of allelic fixation by genetic drift^6^. Deleterious mutations are hypothesized to be the genetic basis of inbreeding depression and heterosis in other major crops including maize and cassava that have outcrossing mating systems^7^. Thus, understanding the genome-wide patterns of deleterious mutations across the wild-to-domesticated continuum of soybean can help to better optimize soybean breeding and potentially other major crops.

After the release of the draft soybean genome sequence^8^, efforts to characterize soybean genetic variation by single-nucleotide polymorphism (SNP) array genotyping^2,9^ and whole-genome resequencing (WGS)^10–14^ have resulted in the global catalog of common and rare SNPs across the genome. However, those data have yet to be fully utilized in an integrated manner to impute marker genotypes at millions of SNP loci as performed for other plant species^15–17^. In addition, the genetic variation of wild soybean remains largely untapped and unexplored relative to that of domesticated soybean.

Here, we analyze the genomic variation of 781 soybean individuals consisting of 418 *G. max*, 345 *G. soja*, and 18 hybrid (*G. max* × *G. soja*) accessions obtained through high-coverage (>13X) WGS data. We conduct the detection of presumed domestication-selective sweeps and the identification of putative deleterious mutations in soybean populations. We then show the usefulness of our data in genetic mapping by imputing millions of the identified SNPs to a panel of 8844 soybean accessions^9,18^ for enhancing genome-wide association studies (GWAS) of seed protein and oil traits.

## Results

### Genomic variation

We collected WGS data for a total of 855 samples from 833 soybean accessions that cover the worldwide distribution of soybean while including large regional collections from Korea, a central region in the geographic distribution of indigenous soybean^2^ (Supplementary Data 1 and Supplementary Note 1). The 855 samples included 22 replicated samples that were added to examine the cause of high heterozygosity rate in some samples observed at the initial stage of this study. Of the 855 samples, 74 that showed higher than two-thirds of heterozygous to homozygous non-reference SNP ratios or an inbreeding coefficient per individual of <0.8 were excluded from further downstream population analyses (Supplementary Figs. 1 and 2). The final non-redundant 781 accessions comprising the haplotype map panel consisted of 418 *G. max* including 332 landraces and 86 improved lines, 345 *G. soja*, and 18 natural hybrid (*G. max* × *G. soja*) accessions. The *G. soja* and hybrid accessions were obtained from China, Korea, Japan, and the Russian Far East. The sequence data of the 781 accessions were mapped to the soybean Williams 82 reference genome ver. Wm82.a2.v1^8^ with mean depths ranging from 14.09 to 61.27 after removing duplicate reads and covered over 95.2% of the reference genome by more than one read and over 85.4% by more than five reads for all accessions whose lower bound of coverage is >10% higher than those of rice and maize^19,20^. After variant calling and filtration steps, we retained 10,597,683 high-quality SNPs to perform most of the population analyses, with the exception of the mutation load analysis that used 30,753,511 SNPs without the 1% minor allele frequency (MAF) filter (Supplementary Fig. 3, Supplementary Table 1, and Supplementary Note 1). Of the identified indels, 1,436,499 indels (17% of raw calls) were used for population analyses. The indels were then separated into 1,414,161 small indels (≤50 bp) and 22,338 structural variants (SV) (>50 bp) (Supplementary Fig. 4). The false-positive error rate of variant calling, estimated with the proportion of segregating sites in the reference accession, was <0.01% (Supplementary Fig. 5 and Supplementary Note 2). By comparison to the 180K SoyaSNP array data^2^, we estimated the power to detect SNPs at 1% MAF for samples in our study is >99% across the genome (Supplementary Fig. 6 and Supplementary Note 2).

### Population structure and diversity patterns

**Fig. 1:**
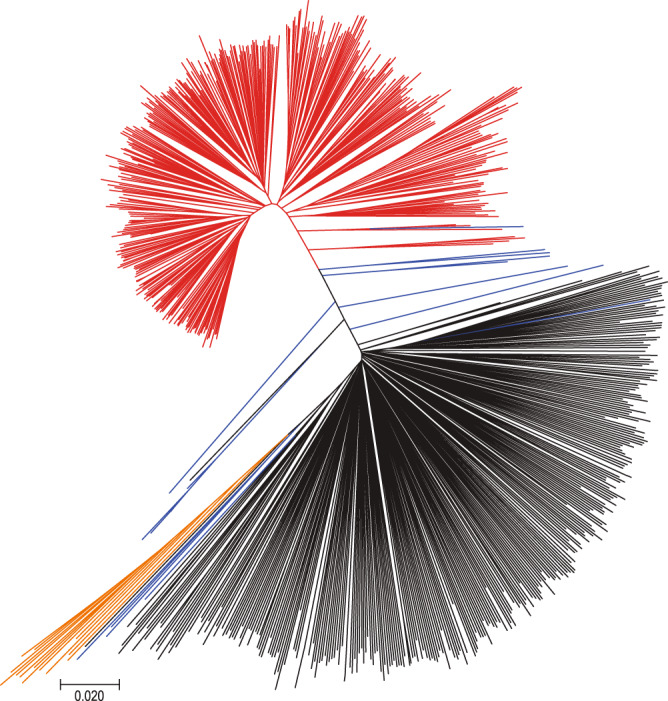
Neighbor-joining tree of the 781 soybean accessions. The accessions were divided into four color lines: *Glycine max* is red, most of *Glycine soja* black, *G. soja* collected from the middle region of the Yellow River basin orange, and hybrids blue.

The population structure of the 781 soybean set assessed using the 10.6 million SNPs (Supplementary Figs. 7 and 8, Fig. 1, and Supplementary Note 3) was similar to that from our recent analysis of 3036 non-redundant soybean accessions genotyped using the 180K SoyaSNP array^2^. However, unlike the tree topology constructed from 180K SNP array data that had some ascertainment bias that favored selection of *G. max* soybean SNPs^21^, branch length differences between *G. max* and *G. soja* in our phylogenetic tree (Fig. 1) reflected almost two times higher nucleotide diversity (*π*) in *G. soja* (0.0023) than *G. max* (0.0012) in our 781 soybean genome population with a *G. soja* level (0.0020) in hybrid samples. The results indicate that, consistent with previous observations^12,22^, roughly half of the genetic diversity has been lost during domestication from wild (*G*. *soja*) to domesticated soybean, which supports the occurrence of a bottleneck in the genetic pool during the soybean domestication process.

**Fig. 2:**
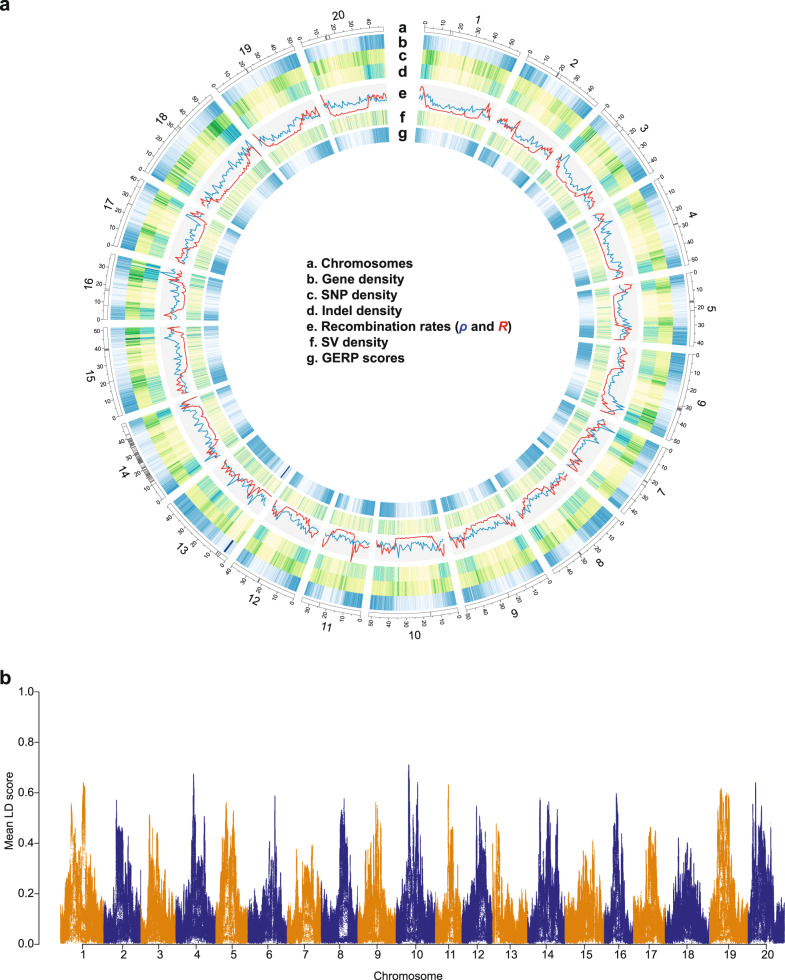
Genomic landscape of soybean. **a** Chromosomes based on the Williams 82 reference genome sequence v. Wm82.a2.v1 (a). Centromere repeat regions are indicated by gray bands. Gene density heatmap (b). SNP density (c). Indel density (d). Population recombination rates calculated in 1 Mb windows (blue = historical recombination rate and red = estimates of recombination rate from mapping populations (e). SV density (f). Average GERP score density (>0), with dark blue of high GERP score (g). All window sizes are 100 kb except recombination rates. **b** Mean LD scores estimated with a 1 Mb window.

Genome-wide profiling of variants was performed on the Williams 82 reference genome to reveal diversity patterns in soybean (Fig. 2). Historical recombination rates (*ρ*) varied substantially along chromosomes, consistent with observations in other plants^23,24^. All chromosomes had lower recombination near the centromere repeat regions, which are presumed to be within pericentromeric regions spanning more than 10 Mbp, relative to that in euchromatin regions. This pattern of recombination frequency distribution has been well supported experimentally by studies of multi-parental maize mapping populations^23,25^, although recombination rates were detected to be almost entirely suppressed in pericentromeric regions in those mapping populations. With available estimates of the recombination rate (*R*) from four soybean inter-crossed bi-parental populations, which captured ~38,000 meiotic crossovers^26^, we compared our estimates of historical recombination rates with empirical estimates of the recombination rate. Overall, *R* and *ρ* were moderately correlated, suggesting that our historical recombination rate estimates inferred on the basis of the SNP distribution likely reflected naturally occurring recombination patterns (Spearman’s correlation coefficient = 0.256, *P* = 7.945e−16).

The overall chromosomal distribution patterns of gene density, SNP density, indel density, and genomic evolutionary rate profiling (GERP) scores were similar to those of recombination rates (Fig. 2). A detailed description of GERP scores is provided below. The patterns of these variables we observed across the genome were significantly correlated, with the strongest correlation between gene density and GERP score density (Supplementary Table 2) as has been reported in other plant genomes^27,28^. We then estimated the patterns of linkage disequilibrium (LD), which is strongly influenced by the mutation and recombination history among many factors. LD (*r*^2^) decay was faster in *G. soja* than *G. max* (Supplementary Fig. 9). LD decreased to half of its maximum value at ~11 kb in wild soybean (*G. soja*, *r*^2^ < 0.2 within 9 kb), similar to those of a previous soybean study^12^ and outcrossing wild rice (*Oryza rufipogon*, ~20 kb) including both perennial and annual forms^29^. In contrast, the LD decay was much slower than that of annual outcrossing wild maize (*Z. mays ssp. parviglumis*, *r*^2^ <0.2 within 0.1 kb)^30^. In domesticated soybean, LD increased to 97 kb similar to that of predominantly selfing cultivated rice (~123 and ~167 kb in *indica* and *japonica*, respectively)^31^ but much higher than outcrossing cultivated maize (*r*^2^ < 0.2 in 5.5 kb)^19^. We found that the local LD of pericentromeric (heterochromatic) regions with ~97 kb of half LD decay distance was much greater than that of euchromatic regions with ~7 kb in total population (Fig. 2b and Supplementary Fig. 9). In each of the subpopulations, the half LD decay distances of heterochromatin regions were at least >11 times greater than those of euchromatic regions. Thus, the chromosomal distribution pattern of LD is negatively correlated with historical recombination rate, gene density, SNP density, indel density, and GERP score (Supplementary Table 2).

### Signals of selection for domestication in soybean

**Fig. 3:**
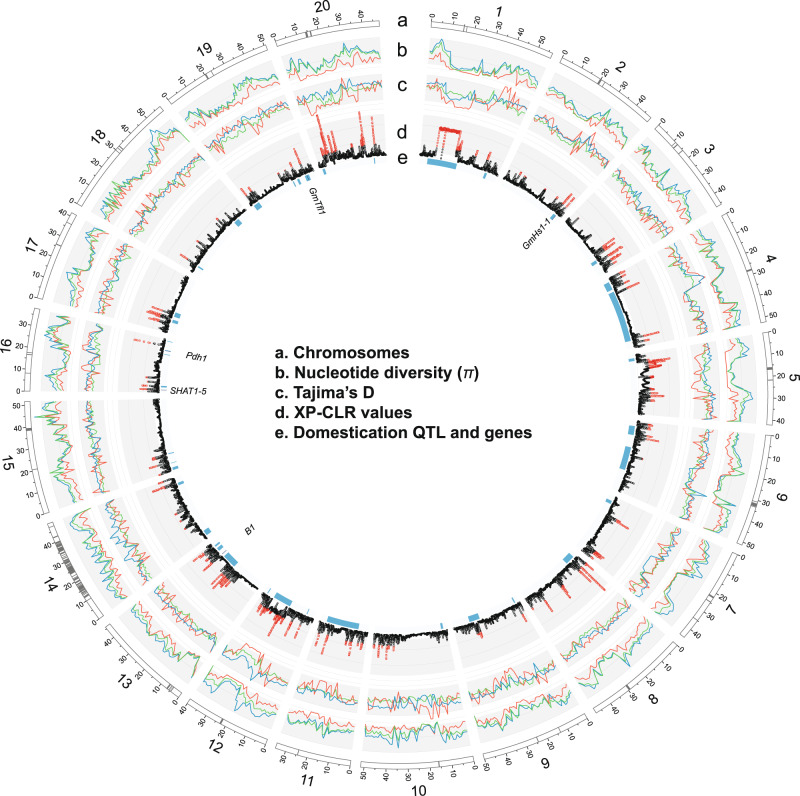
Genomic landscape of selection signals for domestication in soybean. Chromosomes based on the Williams 82 reference genome sequence v. Wm82.a2.v1 (a). Centromere repeat regions are indicated by gray bands. Nucleotide diversity (*π)* in 1 Mb windows for each soybean subpopulation (red=*G. max*, dark blue=*G. soja*, green=hybrid) (b). Tajima’s D for each soybean subpopulation (red=*G. max*, dark blue=*G. soja*, green=hybrid) (c). Distribution of genome-wide likelihood (XP-CLR) values for selection during domestication (d). Plot is based on XP-CLR scores of 100-kb block with 10-kb sliding windows. Domestication quantitative trait loci (QTL) and genes on chromosomes as detected in a large mapping population Williams 82 × PI 479752^33^ (QTL = blue bands and genes = dark blue bands) (e). Gene names are also shown.

Our dataset derived from a collection of 418 domesticated accessions and a comparable number of wild accessions provides an enhanced opportunity for the scanning of selective sweep regions during domestication in soybean. To identify potential selective signals during soybean domestication (wild versus domesticated soybean), we scanned genomic regions with extreme allele frequency differentiation over extended linked regions using a likelihood test (the cross-population composite likelihood ratio, XP-CLR)^32^. A total of 183 domestication-selective sweep regions were detected (Fig. 3). Selective sweep regions had a mean size of 368 kb containing an average of 20 genes and accounted for 6.4% of coding sequence (CDS) in the soybean genome (7,215,740 bp of CDS for selective sweeps versus 104,886,718 bp CDS for the rest of the genome). The detected selective sweeps showed multiple signatures of selection, including elevated differentiation and an expected profile of nucleotide diversity reduction in domesticated soybean relative to wild soybean (Fig. 3). More selective sweep regions were detected on chromosomes 3, 5, 11, 13, and 20, consistent with the previous results that used small numbers of wild soybean accessions^12,13^. A notable exception is two adjacent large selective sweep regions spanning roughly 13 Mb at the pericentromeric region of chromosome 1. In this region, both domesticated and wild soybean had low nucleotide diversity reflecting a general pattern of pericentromeric regions in plant genomes. However, Tajima’s D values for the domesticated soybean population were highly negative, indicating that this large pericentromeric region might have rapidly accumulated rare alleles after selection of key loci for domestication.

When peaks on soybean chromosomes identified as putative selective sweeps were compared with domestication-related QTL from a recent comprehensive study using soybean bi-parental (domesticated × wild) populations (Fig. 3 and Supplementary Note 4)^33^, the comparison supported the selective sweeps identified in this study. Out of 42 chromosomal regions containing unique and overlapping QTL, about 70% corresponded to chromosomal regions detected by XP-CLR. Among 17 QTL that had more than 5% of phenotypic variance explained, 13 corresponded to the selective sweep regions that were detected by XP-CLR. However, because several QTL spanned more than 20 Mb around pericentromeric regions that have low recombination rates, these comparisons should not be considered conclusive but rather suggestive of findings for further study. Two (*GmHs1-1* and *Bloom1*) of several genes implicated in having involvement in soybean domestication were supported by XP-CLR scores and frequency comparison of major domesticated alleles as weak domestication genes or hitchhikers (Supplementary Data 2 and Supplementary Note 4).

### Reduced genetic load in selective sweep regions

**Fig. 4:**
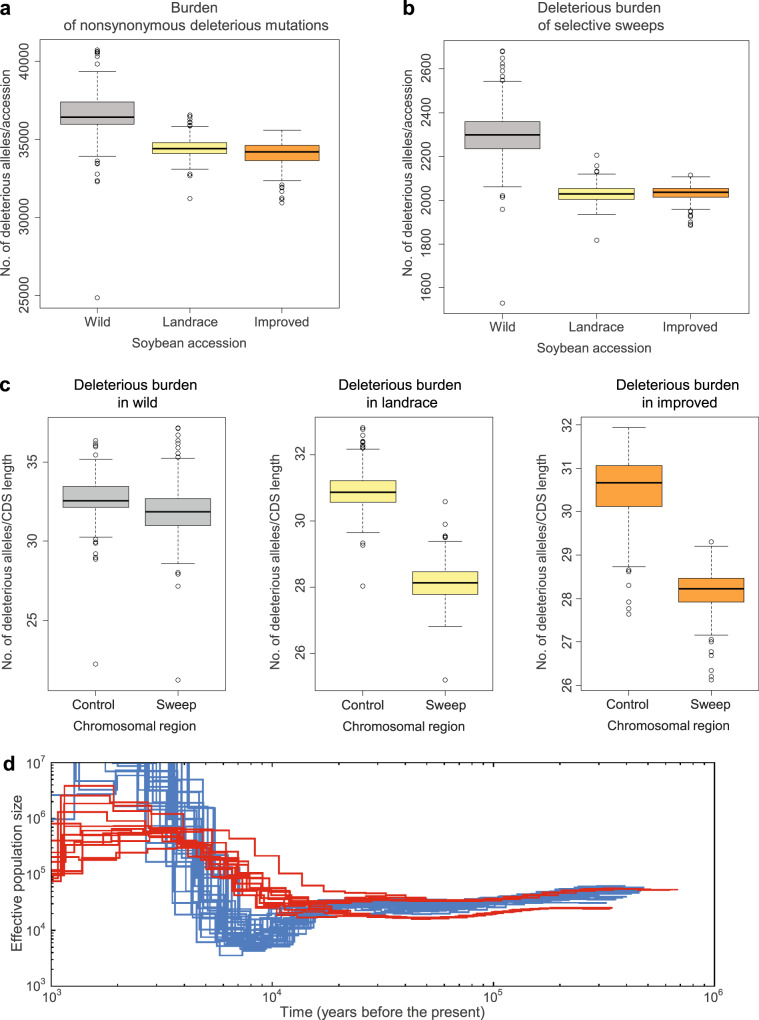
Box-and-whisker plot distributions of mutation burden in domesticated and wild soybean populations and demographic history of soybean. Each box represents the median and interquartile range (IQR). The whiskers represent the range of 1.5 times IQR and the open circles beyond the whiskers are outlier values. **a** Total mutation burden in individual domesticated (*Glycine max*, landrace cultivars = 332 and improved lines = 86) and wild (*Glycine soja*, *n* = 345) soybean accessions. **b** Mutation burden among landrace, improved, and wild soybean accessions in domestication sweep regions. **c** Mutation burden in wild, landrace, and improved soybean accessions between domestication-selective sweeps and control regions (rest of the genome). Vertical axis shows number of deleterious alleles per 100-kb CDS length. In panels **a**–**c**, the subgroups in each of plots are significantly different between one another with *P* < 2.2e−16 in two-sided *t*-tests or Tukey multiple comparison tests except deleterious burdens between landrace and improved soybean accessions with *P* = 0.0003 (**a**) and with *P* = 0.97 (**b**) and deleterious burdens with *P* = 5.4e−13 for deleterious burden in wild soybean accessions (**c**). Source data are provided as a Source Data file. **d** PSMC-inferred demographic history of domesticated (blue lines) and wild (red lines) soybean. Each line represents the change in the past effective population size through time inferred for a pair of genomes. Both the *x* and *y* axis are log_10_ scaled.

Deleterious alleles that are tightly linked to the strongly selected allele in selective sweeps may be less effectively purged relative to those on neutral backgrounds. Studies with several predominant or obligate outcrossing species^27,34–36^ showed that the process of domestication has resulted in an increased number of deleterious variants in the domesticated genome, supporting the cost of domestication hypothesis^37,38^. Here, to quantify the extent of purifying selection on deleterious alleles in the self-compatible, predominantly selfing plant soybean, we used GERP scores^39^. GERP scores were obtained by computing constraint for individual positions across the soybean genome on the basis of comparative genomic approaches using the whole-genome sequence alignment of 12 species including six species in the family Fabaceae. GERP identified 237.5 Mb of the soybean genome (24.3%) as evolutionarily constrained (GERP > 0), and 111.5 Mb (11.4%) as highly evolutionarily constrained with GERP > 2, which is frequently used as a cut-off GERP score to identify the deleterious mutations in constrained portions of the genome in previous studies^27,34,40^ (Supplementary Fig. 10). As expected from the distribution pattern of GERP scores on chromosomes (Fig. 2), we found that 41.1% of the 1,187,829 total SNPs and 48.9% of the 742,149 nonsynonymous SNPs including stop mutations inside CDS were also highly evolutionarily constrained (GERP > 2) in soybean. As a result, we defined a set of 742,149 deleterious mutations with GERP > 2 for exploring the mutation burden in domesticated and wild soybean populations. To examine the impact of the improvement process, the domesticated soybean accessions were further divided into landraces and improved lines. To allow for a comparative analysis, we used *Phaseolus vulgaris* and *Vigna radiata* genomes, which diverged from soybean 19 million years ago^41^, as outgroups to identify derived deleterious alleles in soybean. Results showed a 7.1% decrease (*P* < 2.2e−16) of overall deleterious alleles in landraces relative to wild soybean accessions and 1.4% additional decrease (*P* = 0.0003) in improved lines (Fig. 4a).

In comparisons between domesticated and wild soybean accessions, we found that domesticated soybean showed 11.7% (landraces) and 11.8% (improved lines) fewer (*P* < 2.2e−16, Fig. 4b) deleterious alleles in sweep regions. Thus, the decrease in deleterious alleles has likely been enhanced by artificial selection, suggesting the decreased mutation load we observe in soybean has been driven by reduced hitchhiking of deleterious alleles in linkage regions associated with the selection of specific genes. However, total mutation burden between landraces and improved lines was significantly different (*P* = 0.0003, Fig. 4a) while there was no significant difference between the two groups in selective sweeps (*P* = 0.97, Fig. 4b), indicating improvement selection outside of selective sweeps during modern soybean breeding. In addition to the comparison between the domesticated and wild populations, within-population comparison of sweep regions with the rest of the genome in deleterious alleles showed that selective sweeps exhibited 9.0% (landraces) and 7.6% (improved lines) decreases (*P* < 2.2e−16, Fig. 4c) in deleterious alleles in domesticated soybean. However, in wild soybean, levels of deleterious alleles in regions corresponding with selective sweeps were only slightly lower (2.4% decrease of mean) than those in the rest of the genome (*P* = 5.4e−13, Fig. 4c). Collectively, these results suggest that haplotypes containing fewer deleterious alleles have been favored during artificial selection.

Patterns and levels of extant genetic diversity in crop plants are strongly influenced by domestication history^42^. We inferred the demographic history of the soybean populations using the pairwise sequentially Markovian coalescent (PSMC)^43^. Because both domesticated and wild soybean are a predominantly selfing species, we adopted a strategy of creating pseudodiploid genomes from data for two individuals, similar to what has been done in other inbreeding species such as African rice^44^ and *Caenorhabditis*^45^. Using this approach, we found that domesticated soybean experienced a continual reduction of effective population size (*N*_e_) starting ~15,000 years ago until its nadir from ~5000 to 9000 years ago (Fig. 4d), which corresponds to the time of domestication^1^, and is followed by rapid population growth. The bottleneck sizes ranged from 9000 to a minimum of 3500. However, no severe bottleneck was evident in wild soybean. This severe bottleneck during the domestication of soybean is similar to those observed in other annual crop species^35,36,44,46^.

Although we used a set of nonsynonymous SNPs with GERP > 2 to estimate mutation load, there were ~0.4 million synonymous SNPs with GERP > 2 detected in CDS in this study. Even though synonymous SNPs are largely ignored to be deleterious in mutation load studies, numerous studies have shown that codon bias, which refers to the uneven use of synonymous codons in the transcriptome, serves as a secondary genetic code^47,48^. A GERP score of 4 was also suggested as a stricter cut-off for deleterious alleles^34,40^. When we estimated mutation load using SNP subsets divided by these criteria such as nonsynonymous vs. synonymous and 2 < GERP < 4 vs. GERP > 4 and then compared mutation burden among landrace, improved, and wild soybean populations, the results showed that the overall difference in patterns of mutation load were the same as those from the criteria of nonsynonymous SNPs with GERP > 2 with different percentages of differences (Supplementary Fig. 11).

We also estimated the mutation burden in domesticated and wild soybean populations using 315,029 nonsynonymous deleterious SNPs defined by SIFT score <0.05 with the correction of reference bias (Supplementary Fig. 12)^49^. We observed similar patterns in the mutation load among subgroups to those of GERP-based estimation (Supplementary Fig. 13). However, even with the correction, an ~37–68% additional decrease of overall deleterious alleles in domesticated relative to wild soybean accessions were observed. Interestingly, the mutation load in Williams 82K, a variant of the soybean reference Williams 82, based on the SIFT scores was the lowest among all the estimated soybean accessions, which is similar to the results of a recent report that the number of SIFT deleterious alleles of the reference genomes were the lowest among the estimated 15 barley and eight soybean accessions^50^, thereby indicating that the reference bias correction was not sufficient. However, the mutation load in Williams 82K based on the GERP scores for which we omitted the soybean reference genome when computing the GERP scores to prevent any reference bias was the fifth smallest among all the estimated soybean accessions. These results are somewhat consistent with a previous study^51^ that SIFT is more susceptible to reference bias compared to the other approaches including GERP. This notion was further supported by our observation of properties of candidate domestication genes whose nonsynonymous alleles have been almost fixed in the domesticated population (allele frequency >0.99) and rare in the wild population (allele frequency <0.01) (Supplementary Data 3 and Supplementary Note 5). We collected 29 such candidate domestication genes, two of which contained multiple such SNPs. Chromosomal locations of the candidate genes collected supported our XP-CLR results because 24 of the 29 genes located at the selective sweeps identified in this study and the other five located within 200 kb from the identified selective sweeps. However, of the 29 genes, 26 were predicted to contain tolerated SNPs with SIFT scores >0.05. In other words, the tolerated alleles of the 26 genes that have likely been under selection were not used to estimate the mutation load defined by SIFT score. However, 21 of the 29 genes had GERP scores >2, which is our cut-off score for deleterious alleles, and 24 genes had derived reference allele status.

### Uses of the haplotype dataset for genomic association

A major objective for sequencing a large collection of accessions is to impute genotypes to an even larger panel of diverse accessions for improving the statistical power and resolution of GWAS. We evaluated the usefulness of our generated dataset for GWAS by imputing SNP genotypes to an existing SoySNP50K genotype and phenotype dataset for dissecting the genetic architecture of seed protein and oil content in a large mapping population^9,18^. In soybean, numerous linkage analysis and GWAS efforts have been conducted for these two important traits^52,53^.

**Fig. 5:**
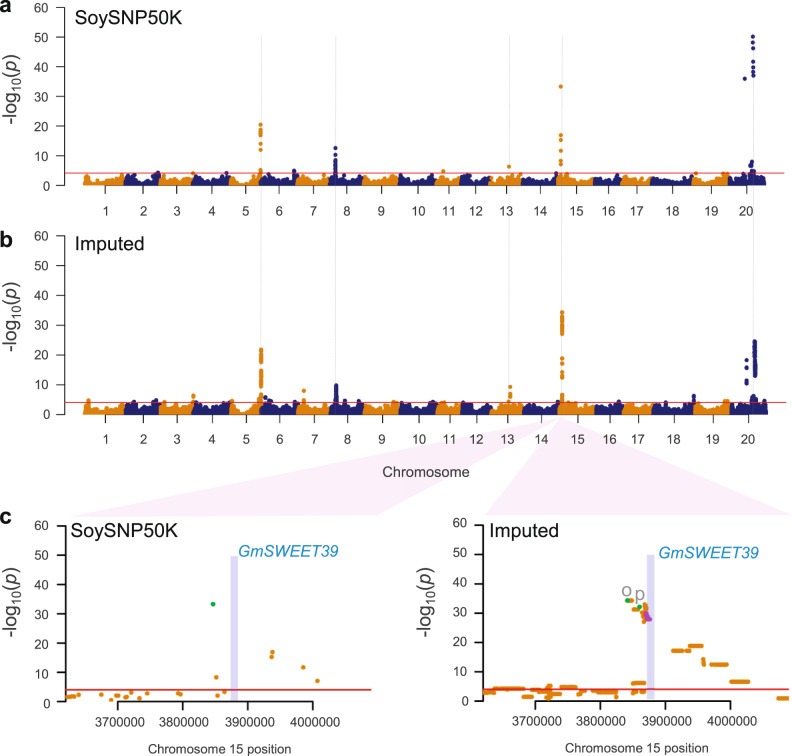
Comparison of mvMLM-based GWAS for oil and protein contents using unimputed and imputed genotype data. **a** Results using the original genotype data from SoySNP50K array. Horizontal red line represents 5% significance thresholds corrected for multiple testing using Benjamini–Hochberg. Five major peaks are indicated by dashed vertical lines for comparison. **b** Results using 3.1 million SNP imputed data that imputed SNP data from 418 soybean genomes into SoySNP50K data. **c** Comparison of mvMLM-based GWAS results using unimputed (SoySNP50K) and imputed genotype data at a major peak on chromosome 15. A pale blue box indicates a chromosomal region of oil content regulator GmSWEET39 that includes its genic region and 5 kb of each of its 5′ upstream and 3′ downstream regions. SNPs located in the *GmSWEET39* region are highlighted by purple dots. A single peak SNP for the SoySNP50 data and unique single peak SNPs for each of protein (p) and oil (o) for the imputed data identified from the MLMM analysis are also highlighted by green dots.

We re-analyzed the previous GWAS of seed protein and oil because of a substantial update of the soybean reference genome version and to eliminate many nearly identical accessions in the original 12,116 soybean accession set (Supplementary Figs. 14 and 15, Supplementary Table 3, and Supplementary Note 6). We then imputed 4,467,134 SNP genotypes to 8844 non-redundant soybean accessions with 36,489 SNPs from the SoySNP50K data (Supplementary Fig. 16 and Supplementary Note 7). The 3,082,234 SNPs with accuracy of median Beagle *r*^2^ of 0.95 after filtration based on the imputation accuracy assessment were used for GWAS. The GWAS results of the two traits from conducting a linear mixed model (LMM) with the imputed SNP dataset on the 8844 accessions were quite similar to our re-analysis results based on the existing SoySNP50K array genotype and phenotype dataset (Fig. 5 and Supplementary Fig. 17). As expected, major peaks were identified for both seed oil and protein. Interestingly, more than 10 novel minor significant peaks such as those on chromosomes 2, 4, and 10 appeared for each of the oil and protein traits and in multivariate GWAS. Although they were clearly found, not a single SNP at these regions reached genome-wide significance in the previous GWAS with the SoySNP50K genotype dataset^18,53^. However, when we performed a multi-locus mixed-model (MLMM) analysis on the same dataset, none of the novel minor signals remained significant (Supplementary Fig. 18). This suggests that the minor signals are very weak effects not retained in the optimal model or the result of complex long-range LD patterns.

For the sake of simplicity for examining any improvement of our imputed GWAS, we focused on five significant major peaks on chromosomes 5, 8, 13, 15, and 20 from multivariate LMM (mvLMM) (Fig. 5a), which were supported by both the LMM and MLMM approaches. Similar to the previous GWAS that used imputed datasets^17,54,55^, the general width and shape of the peaks detected from unimputed data remained largely the same as those from the imputed data with slightly more dense and broader peaks (Fig. 5b and Supplementary Fig. 19). The number of significant SNPs increased, and the most significant SNPs showed improvement in signal strength and shifted in position in GWAS with imputed data with a notable example of the major peak on chromosome 13. Among genes that have been reported as regulatory genes for oil content in soybean, the *GmSWEET39* (*Glyma.15g049200*) gene provided an opportunity to examine the improvement of our imputed GWAS. *GmSWEET39* was cloned as a gene controlling seed oil content by selection during soybean improvement and was suggested as the causal gene for the major association peak on chromosome 15^56^. The most significant SNP did not shift in a notable manner (Fig. 5c) and the SNP is not located at the genic region of *GmSWEET39*. The most significant SNP does not necessarily correspond with variants from a causal gene of an association peak, as notably shown by a rice GWAS^57^. Interestingly, the *GmSWEET39* (Gm15:3,875,081..3,876,544) gene is located in a newly observed association region (~7.0 kb) with 34 significant imputed SNPs (higher than –log_10_(*p*-value) of 27.86) in the middle of the chromosome 15 peak, which was non-significant valley in the GWAS with the original unimputed data (Fig. 5c). Because the *GmSWEET39* gene would not have been regarded as a candidate causal gene in the GWAS with unimputed data, this observation serves as apparent evidence that GWAS with imputed data had the potential benefit of better pinpointing candidate causal genes in soybean.

## Discussion

The discovery and characterization of extensive genome-wide genetic variation in the 781 diverse soybean accessions containing an enhanced number of wild soybean accessions provided us with an opportunity to find unique features of plant genomes that were largely due to both wild and domesticated species being predominantly self-pollinating. The most striking feature is that mutation burden was reduced in domesticated relative to wild soybean accessions. During the past decade, studies of deleterious alleles identified from genome-wide fine genetic variation data of major crops including rice, maize, sorghum, cassava, and grape as well as of dogs have revealed that more deleterious alleles have remained in their domesticated accessions^27,34–37,58^ (see ref. ^42^ for a review) except a recent report of sorghum^28^. However, those well-characterized major crops have different reproduction modes from soybean. Both wild and domesticated species of maize are predominantly outcrossing. Domesticated species of rice and sorghum tends to be selfing while their wild types are predominantly outcrossing. Both wild and domesticated species of cassava and grape are outcrossing; however, cultivated types are predominantly clonally propagated. Nonetheless, genome-wide patterns of soybean variation were similar to those of other major crops including the well-characterized maize, although the genome-wide nucleotide diversity and half LD distance estimates appeared to be unique in soybean. Moreover, soybean showed a similar demographic history as the other major crops investigated. Our results of total mutation burden comparison are in contrast to the previous studies from cassava, grape, maize, rice, and sunflower^27,34–37,58^. Interestingly, our results are similar to a substantial decrease of the homozygous-mutation burden in domesticated cassava and grape accessions, compared with progenitors^27,36^. In selfing taxa *Arabidopsis*, selective sweeps of extreme haplotype sharing were observed likely due to removal of variation^59^. Decrease of overall mutation burden from landraces to improved lines, which are inbreds, was also observed between inbred elite maize lines and their comparable landraces^19,60^. This decreasing trend of the homozygous-mutation burden somewhat reflects the behavior of recessive model for human populations that leads to a slight decrease in recessive load^49^. Taken together, our results suggest that the selfing reproduction mode of both the domesticated and wild forms contributed to reduction in mutation burden in domesticated relative to wild soybean accessions^5^.

The findings from this study may be extended to the characterization of wheat and barley^61,62^, which have the same reproduction mode as soybean but whose genome analyses have lagged behind due to their huge genome sizes. While there has been the lack of quantitative comparisons of deleterious alleles between domesticated and wild populations of these crops, deleterious alleles were identified from 8 soybean and 15 barley accessions and then their enrichment within genes associated with phenotypic traits were shown^50^. For wheat, exome capture sequence data of 890 diverse landrace and cultivar accessions were collected and the identified variants revealed the reduced deleterious allele burden by introgression and selection for improvement and environmental adaptation in cultivars compared with landraces^63^.

Of the originally resequenced 855 samples, we excluded 74 samples (8.65%), which showed high heterozygosity and low inbreeding coefficient, based on the presumed reproduction mode of soybean. The 781 soybean accessions were clearly divided into domesticated and wild accession groups with a distinct subgrouping of wild accessions according to geographic collection sites, in a similar fashion to other major crops. However, compared to maize landraces that showed only 17% diversity reduction from their wild progenitor^64^, a drastic reduction in nucleotide diversity (∼48%) was observed during the transition from wild to domesticated soybean. This likely reflects different reproduction modes between selfing soybean and outcrossing maize. The overall chromosomal distribution patterns of variation of several variables including recombination, gene density, and LD were also quite similar to those observed in other major crops.

A diverse collection of 345 wild soybean accessions were analyzed against 418 domesticated accessions to detect selective signals for soybean domestication. Although many canonical domestication genes have been cloned from major grass crop species, such knowledge has not been translated well to domestication research in eudicot seed crop species including soybean. In major crop species, cultivated species and their progenitors usually show distinct morphological and physiological differences in so-called domestication syndrome traits such as seed size, shattering, seed dormancy, flowering time, and viny growth habit. Soybean is not an exception. However, organs and tissues where several domestication traits are expressed differ between soybean and grasses. For example, shattering is related to the pod in soybean, but to pedicel in rice. Unfortunately, our analysis shows that although two of them might be regarded as weak domestication genes, none of the soybean domestication genes cloned thus far should be regarded as a canonical domestication gene. However, in this study, we reported many candidate canonical domestication genes whose alleles are almost fixed in domesticated soybean and are rare in wild soybean.

In this selfing species, overall deleterious alleles among landraces relative to wild soybean accessions, which were defined by GERP scores, have been moderately reduced by up to almost 7%, similar to the observation in sorghum^28^. Mutation burden was further decreased in improved lines from modern soybean breeding. The results are in stark contrast to the observations that deleterious alleles (the genetic load) that happen to be present in the neighborhood background of the strongly selected allele in the presence of selective sweeps may become more prevalent than those in other neutral backgrounds^37,38^. However, a direct comparison in terms of the accumulation of deleterious alleles may be difficult because of different reproduction modes between soybean and other well-studied crops. Purging of deleterious alleles from the domesticated soybean has been further enhanced in selective sweep regions. Unlike outcrossing species that maintained accumulated deleterious mutations in the heterozygous state, this predominantly selfing plant may have been less tolerable to the accumulation of deleterious alleles, eventually leading to the reduction of diversity. Introgression of untapped variation in wild soybean should be an important objective for the future breeding of soybean. Most of the deleterious alleles should be prioritized for elimination through breeding process, although a handful of deleterious alleles may provide beneficial effects for soybean growth or yield in crop fields. Information obtained here should help better design crossing and selfing efforts to efficiently eliminate deleterious alleles in a breeding program to select agronomically important untapped genes from wild soybean.

Finally, we have shown that our high-quality map of SNP variation in soybean could be used as a reference panel for the imputation of genotypes to improve GWAS of oil and protein traits. In addition to those unique genome variation features due to selfing and being a eudicot seed crop species that suggest soybean as a model for other such crops, our imputation results suggest that the soybean variation map and methods developed here can be used in a direct manner to accelerate genetic variation discovery in this economically important crop.

## Methods

### Plant materials and sequencing

We initially selected 818 accessions based on the 180K Axiom^®^ SoyaSNP array genotyping data of ~4400 diverse soybean accessions, most of which were collected from South Korea. These diverse soybean accessions also contained representatives from the worldwide distribution of soybean^2^. Soybean plants were grown in the Ochang field of the Korea Research Institute of Bioscience and Biotechnology, Cheongju, Korea. Although more than two times of single plant selection for the SoyaSNP array genotyping had been performed^2^, we collected young leaves from a single plant of each accession and then extracted genomic DNA using the cetyltrimethylammonium bromide (CTAB) method^65^. DNA sequencing was performed at LabGenomics (Seongnam) or Macrogen (Seoul) companies in Korea. Paired-end sequencing libraries were constructed with an insert size of 500 bp using a TruSeq DNA PCR-Free kit (Illumina, San Diego, CA, USA) according to Illumina library preparation protocols. Libraries were then sequenced using Illumina HiSeq 2500 or 4000 platforms with 2 × 151-bp paired reads to a target coverage of 10X. Some accessions that showed high heterozygous variant levels were sequenced multiple times. We also added resequencing data of 16 accessions determined in our previous studies^11,66^ except IT182932 that was newly sequenced in the present study. Consequently, resequencing data from a total of 855 samples were used for initial variant calling in this study (Supplementary Data 1).

### Read mapping and variant calling

Short paired-end reads of 855 samples were quality checked using FastQC (version 0.11.3) (http://www.bioinformatics.babraham.ac.uk/projects/fastqc/). We then essentially followed procedures described in the Genome Analysis Toolkit (GATK) Best Practices for data pre-processing and variant calling^67,68^. We used BWA (version 0.1.12) with default parameters except for –M option^69^ to map genomic reads from each accession against soybean Wm82.a2.v1 reference genome assembly^8^. Alignments were further checked for PCR duplicates using Picard tools (version 1.134) (http://picard.sourceforge.net/). We performed sorting operation, base recalibration, per-sample and joint variant callings, and variant filtration using GATK (version 4.0.1.2). Known variant sites for soybean extracted from NCBI dbSNP Build 144 (https://www.ncbi.nlm.nih.gov/projects/SNP/snp_summary.cgi?build_id=144) were used for base recalibration. Raw variant calling data were divided into SNPs and indels with SelectVariants function of GATK (v. 4.0.1.2). A total of 62,987,283 SNPs and 8,567,041 indels were identified from the analyses of the genomes of 855 samples. Quality filtering of raw SNP calls was performed using VariantFiltration in GATK according to the following criteria: ReadPosRankSum of <−2.0, MQRankSum < −2.0, polymorphism confidence scores (QUAL) < 30.0, genotype call quality divided by depth (QD) < 3.0, Phred-scaled *P*-value of Fisher exact test for strand (FS) > 30.0, mapping quality (MQ) < 30.0, total depth of coverage (DP) < 100, genotype-filter-expression depth of coverage (DP) < 5, and genotype-filter-expression genotype call quality (GQ) < 10.0. Bi-allelic variants were then selected using VCFtools (version 0.1.15)^70^. To exclude erroneous variants in repetitive regions, variants with high mapping depth (>4X reads per sample, where X was mapping depth) in each sample were masked. Allele balance (AB) was calculated and variants with AB < 30 in heterozygous genotypes were masked^71^. The VCFtools was then used to remove markers that were monomorphic and markers with call rates <50%. Up to this stage of filtration, 36.8 millions of SNPs were defined as candidate variants. In the 62.9 million raw SNP calls, some samples showed more heterozygous than homozygous non-reference alleles. Those samples still showed high heterozygous rates in the 36.8 million candidate SNP set. Thus, 66 samples that contained higher than two-third heterozygous to homozygous non-reference SNPs ratios among the samples with more than 0.5 million heterozygous SNPs in the raw SNP call set were excluded from further analyses. The inbreeding coefficient per individual was then calculated as the difference between the expected and the observed heterozygosity standardized by the expected heterozygosity under Hardy–Weinberg. Based on the assumption that pure inbred lines would show inbreeding coefficients of near 1.0, we additionally excluded eight wild samples that had <0.8 inbreeding coefficients per individual in the 36.8 million candidate SNP set. Finally, 781 accessions were determined as our soybean genome variation study set. To perform population analyses using a set of 781 accessions, we further filtered these candidate SNPs by removing SNPs with >20% missing calls and >10% heterozygosity and monomorphic SNPs and then removing SNPs with minor allele frequency (MAF) < 1%. Finally, we retained 10,597,683 high-quality SNPs for population analyses of the genomes of 781 accessions except mutation load analysis, which used 30,753,511 SNPs without 1% MAF filtration. Filtering of raw indel calls was performed according to the following threshold criteria: ReadPosRankSum of <−20.0, QUAL < 30, QD < 2.0, and FS > 200. Bi-allelic variants were then selected using VCFtools (version 0.1.15)^70^. A resultant set of 5,717,052 indels was further filtered to obtain high-quality indels using the same cut-off criteria as the SNP filtration. From this analysis of the genomes of 781 accessions, a filtered set of 1,436,499 indels were defined. The indels were then divided into small indels and structural variants (SV) with a cut-off of sequence length of 50 bp.

The Williams 82 reference genome assembly was constructed from sequencing of multiple individuals that contained heterogeneous regions^72^. Genetic polymorphisms identified in Williams 82K, a variant of Williams 82, across the homogeneous chromosomes of the Williams 82 reference genome were used to identify the false-positive error rate of soybean haplotype data. We validated our soybean haplotype data by estimating the concordance rate in genotype calls between the soybean haplotype data and 180K SoyaSNP array datasets^2^.

### Population structure and diversity pattern inference

Principal component analysis (PCA) was conducted to summarize the genetic structure and variation present in the 781 accessions using smartpca function in Eigensoft v7.2^73,74^. We plotted the first three PCs. We further used the model-based, Bayesian clustering software FastStructure v 1.0^75^ to estimate the population structure. FastStructure was run on default settings with 10-fold cross-validation for subpopulations (*K*) ranging from *K* = 2 to 12. Numbers of subpopulations were defined using the marginal likelihood function. We plotted the membership coefficient using DISTRUCT v1.1^76^. A neighbor-joining tree was constructed by MEGA7^77^ under the *p*-distances model.

Nucleotide diversity (*π*)^78^, SNP density, and Tajimas’s D^79^ for 100 kb were calculated with the 10.6 million SNPs using vcftools --window-pi 100000, --SNPdensity 100000, and --TajimaD 100000, respectively^70^. Indel and SV densities for the bi-allelic variants were calculated using vcftools --SNPdensity 100000. Population recombination rates (Rho, *⍴*) were estimated in the entire panel using the machine learning R package FastEPRR v1.0^80^. Linkage disequilibrium (LD) decay was calculated using PopLDdecay v3.31 with -MaxDist 1000 -MAF 0.05 -Miss 0.1 parameters^81^. Measures of LD (*r*^2^) were calculated for each subpopulation using Plot_MultiPop.pl with -bin1 100 -bin2 1000 -break 5000 -keepR parameters implemented in PopLDdecay^81^. The *r*^2^ values were also calculated for the entire population for euchromatic and heterochromatic regions, which were previously defined by the regions with high and low recombination rate^82^. Pairwise LD scores were calculated from the unimputed 10.6 million SNP dataset using the genome-wide complex trait analysis (GCTA) suite (version 1.92.1) with default settings^83,84^. Circos v0.69-6^85^ was used to display distributions of estimated variables on the Williams 82 reference genome ver. Wm82.a2.v1^8^.

### Genome scan for selective signals

To scan selective signals over the soybean genome, we used a widely used cross-population composite likelihood ratio test (XP-CLR) (version 1.0)^32^ updated by Hufford et al.^64^. XP-CLR uses allele frequency differentiation between populations. A total of 763 soybean accessions consisting of 418 domesticated and 345 wild accessions were used for detecting selective sweep regions. Missing variants in our haplotype map data were imputed using the Beagle v5.0^86^ with the default option. Evidence for selection of domestication across the genome was evaluated by comparing domesticated versus wild soybean genomes. Individual SNPs were assigned at positions along with a recombinant inbred genetic map derived from a cross between *G. max* var. Williams 82K and *G. soja* var. IT182932^26^. Markers located on the insertion of unanchored scaffolds or different chromosome segments as well as on chromosome segments whose physical or genetic orders were not collinear between the reference genome and our genetic maps were excluded from the genetic map. Coordinates of the soybean reference genome assembly Wm82.a2.v1 were applied to calculate genetic per physical distance between markers in the genetic map. XP-CLR was performed with the following criteria: -w1 0.0005 200 100 –p1 0.7. In other words, XP-CLR scores of 100 bp windows were calculated for a maximum of 200 SNPs per 0.05 cM genetic window. Markers with a correlation level >0.7 were down-weighted. Manhattan plots of XP-CLR scores were constructed using qqman^87^ in R package or using Circos^85^. Windows with >89.4 of XP-CLR values, accounting for 5% of the genome, were considered as selective sweep regions. Groups of adjacent windows with XP-CLR values not containing more than one window below this threshold were defined as a single sweep region. We assigned the gene closest to the window with the maximum XP-CLR score in each selective sweep region as the most likely candidate.

### Determination of effects of nucleotide variants

To predict functional effects of variants, we used Sorting Intolerant From Tolerant 4G (SIFT 4G)^88^ to annotate the 30.8 million SNP dataset. To create a soybean database, uniref90 (https://www.uniprot.org/, download date: 9 February 2019) was used as a reference protein set. Annotation of *G. max* Wm82.a2.v1 was downloaded from EnsemblPlants (ftp://ftp.ensemblgenomes.org/pub/plants/release-44/gff3/glycine_max). Gff3 format was converted to Ensembl GTF format. Soybean SIFT 4G database was constructed using SIFT4G_Create_Genomic_DB implemented in SIFT 4G. SIFT scores ranged from 0 to 1, and any nonsynonymous position with a SIFT score <0.05 was considered to be putatively deleterious.

### Genomic evolutionary rate profiling (GERP)

We estimated the individual burden of deleterious alleles based on the genomic evolutionary rate profiling (GERP) scores^39^ for each site in the soybean genome. GERP score reflects the strength of purifying selection based on constraint in a whole-genome alignment of multiple plant species. For the whole genome alignment, we used the LASTz/MULTIz approach (http://genomewiki.ucsc.edu/index.php/DoBlastzChainNet.pl) described for the alignment of 20 angiosperm genomes to *A. thaliana* reference^89^ with the following minor modifications. We aligned 12 soft repeat-masked genomes of *Arabidopsis thaliana* (TAIR10.1), *Cajanus cajan* (V1.0), *Lupinus angustifolius* (v1.0), *Medicago truncatula* (4.0), *Oryza sativa* (IRGSP_1.0), *Phaseolus vulgaris* (1_0), *Populus trichocarpa* (v3), *Prunus persica* (v2), *Vigna radiata* (ver6), *Zea mays* (v4) from RefSeq database (https://www.ncbi.nlm.nih.gov/refseq/), and *Vitis vinifera* (V2) from URGI database (https://urgi.versailles.inra.fr/Species/Vitis) to the *G. max* (Wm82.a2.v1) genome using LASTz v1.04.00 and MULTIz v012109. Topology of the 12 species of interest was extracted from the whole phylogenetic tree using ete3 toolkit (v3.1.1)^90^. The phylogenetic tree was downloaded from Phylogenetic Resources files on Dryad database (10.5061/dryad.63q27.2)^91^. The branch length (substitution per site) of the phylogenetic tree was calculated using phyloFit v1.5^92^ with four-fold degenerated sites of chromosome 1 in *G. max*. All alignment files (maf files) were merged using MULTIz and converted to fasta format using maf2fasta function. Alignment gaps (−) in the reference genome (*G. max*) and sequences of the same position in other genomes were removed. Finally, we calculated GERP scores using gerpcol with –j option from GERP++^39^. The -j option projected out the soybean reference sequence to prevent any bias in the estimates using GERP, a method that predicts functional consequences based on phylogenetic information^34,40^. Uncalculated positions were filled with 0 because neither GERP score of N nor n sequence position in *G. max* genome was calculated.

### Mutation load estimation

We estimated genome-wide mutation load using numbers of derived deleterious alleles identified in soybean accessions based on GERP or SIFT scores. From ~30.8 million SNPs, we extracted 1,187,829 SNPs located inside the coding regions of soybean genes (CDS). We then polarized derived and ancestral alleles for the 1,187,829 CDS SNPs using *Phaseolus vulgaris* (1_0) and *Vigna radiata* (ver6) genomes as outgroups. For each variant, the corresponding nucleotides in both the outgroup genomes were identified based on the whole-genome alignment for the GERP score calculation above. We then used the est-sfs (v2.03) software^93^ to infer the probability of the derived versus ancestral allelic state at a polymorphic site. For estimation of GERP-based mutation load, we categorized these mutations into four categories of deleterious variants: nonsynonymous SNPs (stop mutations were included in nonsynonymous mutations in this study) with moderately-conserved deleterious mutations (2 < GERP > 4), synonymous SNPs with moderately-conserved deleterious mutations, nonsynonymous SNPs with highly conserved deleterious mutations (GERP > 4), and synonymous SNPs with highly conserved deleterious mutations. The criterion of GERP > 2 to determine conservative site was proposed by previous studies^27,34,40^ based on the distribution of GERP scores. For most of the mutation load analysis, a combined dataset containing all nonsynonymous SNPs with GERP > 2 was used. For estimation of SIFT-based mutation load, we referred to nonsynonymous SNPs with SIFT score <0.05 as deleterious mutations. However, because we also observed a strong reference bias that sites where the reference soybean genome carries the derived allele are much more likely to be classified as tolerated than are sites where the reference is ancestral as reported in human genetics (Supplementary Fig. 12), we corrected the number of derived deleterious alleles at sites at which the reference genome carries the derived allele by multiplying the estimated probability following Simons et al.^49^. Finally, we summarized the mutation load as the number of derived deleterious alleles in an accession^27,94^.

### Demographic analysis

We conducted historical demographic analysis with PSMC (v0.6.5-r67)^43^, considering each soybean accession as a single genomic haplotype. Because both domesticated and wild soybean are predominantly selfing species, we adopted a strategy of creating pseudodiploid genomes from data for two individuals, similar to those performed in other inbreeding species such as African rice^44^ and *Caenorhabditis*^45^. Eight samples with >19X genome coverage after removing duplicate reads for each of domesticated (*G. max*) and wild (*G. soja*) soybean were used to generate pseudodiploid genomes. Each of SNPs in genomes was called using a SAMtools v1.9^95^ and BCFtools v1.9^96^ pipeline. The soft-masked Williams 82 genome sequence (version Wm82.a2.v1) was used to identify repetitive regions and mask genotype calls overlapping these repetitive regions. Pseudodiploid was generated using Seqtk v1.2 (https://github.com/lh3/seqtk). Heterozygous sites were randomly selected by randbase function and each haploid was merged by mergefa function. The eight samples for domesticated soybean consisted of four (Fu yang (30), No. 39 Green, Ji li huang dou, and PI 72227) from China, two (PI87631-1 and PI87630) from Japan, and two (PI 96786 and PI 458232) from Korea. The eight samples for wild soybean consisted of four (PI 464937 B, PI 447003 B, PI 483464 B, and PI 597459 D) from China, two (B07162 and PI 378691) from Japan, and two (IT182932 and YWS1588) from Korea. Analysis employed default parameters for the PSMC program. Assuming a mutation rate of 1.5 × 10^−8^ mutations per nucleotide per year^97^ and a generation time of 1 year, we converted scaled population parameters into years and *N*_e_. We constructed pseudodiploid genomes for all 28 possible combinations of the eight accessions for each of domesticated and wild soybean. We excluded 15 pseudodiploids of the 28 wild pseudodiploids from further analysis owing to spurious PSMC profiles. Most of the 15 pseudodiploids with spurious profiles were derived from samples within countries. No such spurious profiles were observed from the domesticated pseudodiploids.

### Filtration and imputation of soybean data genotyped using SoySNP50K array

Genotype data in soysnp50k_wm82.a2_41317.vcf that consisted of 42,291 SNPs scored on 20,087 germplasm accessions using the Illumina Infinium SoySNP50K BeadChip^9^ were downloaded from SoyBase as of 10 June 2019^98^. In this dataset, we corrected the genotypes of 3494 reverse-oriented SNP sites in Glyma.Wm82.a2. We removed 96 SNPs presumed to be absent in Glyma.Wm82.a2 because they showed a base that was different from both reference and non-reference bases. We also removed 2 mitochondrial DNA SNPs. The resultant 42,193 then underwent further filtration. From the whole set, we selected a total of 12,116 accessions for GWAS of seed protein and oil content by Bandillo et al.^18^. Of the 12,116 accessions, 559 with heterozygous rate >0.05 or missing rate >0.05 were removed. We calculated identical-by-descent (IBD) values for all pairwise comparisons among 11,557 *G. max* accessions using PLINK v1.9^99^. We considered pairs of accessions to be duplicated if they had an IBD > 0.98^100^. As a result, 3272 duplicates were removed, leaving 8844 non-duplicated accessions with high-quality genotype data. In this set of 8844 accessions, SNPs with heterozygous rate >0.02, minor allele frequency <0.02, and missing rate >0.10 were discarded from the genotype data, leaving a total of 36,489 high-quality SNPs for the imputation of soybean haplotype data and GWAS. Beagle v5.0 was used to impute the SoySNP50K data using 4,467,134 SNP data on chromosomes with MAF > 0.02 from 481 *G. max* genomes. The strategy that we used 481 *G. max* genome data instead of the whole 781 soybean genome is similar to imputation strategies in human genetics that use a part of reference haplotype panels closely related to subjects (e.g., see refs. ^101,102^). A genetic map constructed from a population of 233 recombinant inbred individuals derived from a cross between Williams 82K and IT182932^26^ was used as the fine-scale recombination map input for imputation. Genotype imputation accuracy was assessed in terms of the squared correlation (*r*^2^) between the true allele dosage and the imputed posterior allele probability implemented in the Beagle program^103^. After genotype imputation, we filtered out poorly imputed (Beagle *r*^2^ < 0.3) and low MAF (<0.01) SNPs, resulting in 3,082,234 SNPs available for GWAS analysis.

### Genome-wide association analyses for oil and protein contents

For GWAS for seed protein and oil content on the 8844 accessions using the original SoySNP50K data, of the 36,647 high-quality SNPs, 36,498 SNPs located on 20 soybean chromosomes were used. GWAS on the 12,116 accessions originally used by Bandillo et al.^18^ was also conducted for a comparison using 37,142 SNPs located on the 20 soybean chromosomes with frequency >2%. Missing variants were imputed using Beagle v5.0^104^ with default option. We used GEMMA v0.98.1^105^ to infer the correlation between each variant and seed oil and protein content. We first estimated a relatedness matrix from genotypes using the -gk 1 option in GEMMA. Then, we assessed evidence for correlation in a univariate linear mixed model (LMM) framework using the -lmm 4 option. We also assessed evidence for testing marker associations between oil and protein content as well as for estimating genetic correlations between oil and protein content in a multivariate linear mixed model (mvLMM). The Benjamini–Hochberg procedure^106^ was used to account for multiple testing by controlling the false discovery rate (FDR) at 5%. Manhattan plots were constructed to display GWAS results using qqman v0.1.4^87^ in R package. The GWAS procedure for seed protein and oil content on the 8844 accessions using genotype data that imputed 4.5 million SNPs into SoySNP50K array data of 8844 accessions were essentially the same as that for unimputed SoySNP50K data.

A modified genome-wide approach^107^ for implementing a multi-locus mixed-model (MLMM)^108^ to resolve association signals involving large-effect genes was used to further identify SNPs potentially associated with the oil and protein traits. The MLMM method relies on a simple, stepwise mixed-model regression procedure with forward selection and backward elimination while re-estimating the genetic and error variances at each step of the regression. This method may well lead to higher detection power and a lower FDR relative to traditional single-locus approaches. Because the imputed data appeared to exceed the computing power available, we reduced the number of markers by linkage disequilibrium (LD)-based marker pruning in PLINK v1.9 software^99^. Briefly, we pruned markers from imputed data using the --indep-pairwise 100 25 0.99 option in PLINK. This option considers a window of 100 SNPs, calculates LD between each pair of SNPs in the window, and finally removes one of a pair of SNPs if the LD is >0.99. Next, overlapping SNPs between the imputed data and SoySNP50K data that were deleted during pruning were added back to the pruned data, resulting into 291,388 markers for MLMM models.

### Reporting summary

Further information on research design is available in the Nature Research Reporting Summary linked to this article.

## Supplementary information

Supplementary Information

Reporting Summary

Description of Additional Supplementary Files

Supplementary Data 1

Supplementary Data 2

Supplementary Data 3

## Data Availability

Although 16 of the original data (NCBI SRA accession numbers ERX2248648-ERX48662 and ERR953473) have been released in conjunction with prior publications^11,66^, we uploaded raw reads in fastq format for all 855 final accessions to NCBI SRA with SRA accession number PRJNA555366. Large datasets including SNPs, indels, SV calls, SIFT scores, GERP scores, and ancestral state of CDS SNP variants and the source data for Supplementary Fig. 16 are available from figshare repository (https://figshare.com/projects/Soybean_haplotype_map_project/76110). Data supporting the findings of this work are available within the paper and its Supplementary Information files. A reporting summary for this Article is available as a Supplementary Information file. The datasets and plant materials generated and analyzed during the current study are available from the corresponding author upon request. Known variant sites for soybean, uniref90, and annotation of *G. max* Wm82.a2.v1 were downloaded from NCBI dbSNP Build 144 (https://www.ncbi.nlm.nih.gov/projects/SNP/snp_summary.cgi?build_id=144), UniProt (https://www.uniprot.org/), and EnsemblPlants (ftp://ftp.ensemblgenomes.org/pub/plants/release-44/gff3/glycine_max), respectively. Plant reference genome sequences were downloaded from RefSeq database (https://www.ncbi.nlm.nih.gov/refseq/) and URGI database (https://urgi.versailles.inra.fr/Species/Vitis). The phylogenetic tree was downloaded from Dryad database (https://datadryad.org/resource/doi:10.5061/dryad.63q27.2). Source data are provided with this paper.

## Source data

Source Data
