# Supplementary material for: The patterns of deleterious mutations during the domestication of soybean
Source: Nat Commun. 2021 Jan 4;12:97. doi: 10.1038/s41467-020-20337-3 (PMC7782591; doi:10.1038/s41467-020-20337-3)
Supplement: Supplementary file 1 — Supplementary Information [file 41467_2020_20337_MOESM1_ESM.pdf]

Supplementary Information

The patterns of deleterious mutations during the domestication of soybean

Myung-Shin Kim, Roberto Lozano, Ji Hong Kim, Dong Nyuk Bae, Sang-Tae Kim, Jung-Ho Park, Man Soo Choi, Jaehyun Kim, Hyun Choong Ok, Soo-Kwon Park, Michael A. Gore, Jung-Kyung Moon, and Soon-Chun Jeong

Correspondence to: moonjk2@korea.kr (J.K.M); scjeong@kribb.re.kr (S.C.J.)

Supplementary Notes

Supplementary Note 1. Variation calling and exclusion of highly heterozygous genotype samples

Accession information and statistics of genome sequencing analyses for a total of 855 samples from 833 soybean accessions are summarized in Supplementary Data 1. Analysis of sequencing data obtained at the initial stage in this study indicated that 17 soybean accessions including 12 wild accessions contained higher than 0.5 million heterozygous SNPs in the initial, unfiltered SNP call data (Supplementary Fig. 1). Seven of them contained more heterozygous than homozygous non-reference alleles, in contrast to a simple assumption that an inbred line that had gone through at least two generations of single-seed descent for the current study[1] would show a much lower number of heterozygous genotypes than homozygous genotypes. Such high heterozygous rate did not change after further filtering with the initial set of samples. To test whether such results might be due to experimental errors other than the natural conditions of plants, we repeated the sequencing data analysis using 22 samples prepared under different conditions from the 17 soybean accessions (Supplementary Fig. 1). However, the high heterozygous rate did not change except for one single plant and two filial plant bulk trials. The results suggested that those high heterozygous soybean samples resequenced were likely progeny from cross between different accessions, in contrast to the notion that soybean is predominantly selfing. In results, we analyzed a total of 855 samples including 22 repeated samples. The 855 genomes were aligned to the *G. max* cv. Williams 82 reference genome ver. Wm82.a2.v1[2]. Raw variant calling data from the

Genome Analysis Toolkit (GATK) Best Practices[3] contained a total of 62,987,283 SNPs and 8,567,041 indels. In light of the observation of high heterozygosity, of the 855 samples that included duplicated samples, 66 samples from 54 accessions that contained higher than two-thirds of heterozygous to homozygous non-reference SNPs ratios were grouped as high heterozygous samples. GATK quality filtering and further filtration steps including read depth per sample and allele balance reduced the number of candidate SNPs to 36.8 millions. The relative heterozygosity rate in each sample tended to remain to be similar after these filtrations. Distributions of inbreeding coefficient per individuals in subgroups divided by species and heterozygosity indicated that most samples grouped by the high heterozygosity showed an inbreeding coefficient of less than 0.8 before and after filtration of SNPs with inbreeding coefficient per marker less than 0 (Supplementary Fig. 2). However, eight wild accessions, which were not excluded on this criterion, showed inbreeding coefficient per individual of less than 0.8. Thus, these eight accessions were additionally excluded from further population analyses. Thus, we ended up with the 781 non-redundant individuals consisting of 418 *G. max*, 345 *G. soja*, and 18 hybrid (*G. max* x *G. soja*) accessions.

We excluded 74 samples (8.65%) of the originally resequenced 855 samples. The exclusion rate is much higher than natural cross-pollination rates of soybean below 1% revealed by many well-designed experiments (for a review, see Carlson and Lersten (2004); Lu (2004)). However, higher than 5% cross-pollination rates were reported based on a different way of calculation [6] or in a certain environment[7]. Thus, those highly heterozygous accessions may have higher cross-pollination rates than normal or have had higher cross-pollination rate in a habitat with frequent visits by pollinating insects.

To perform most of the population analyses using a set of 781 accessions, we further filtered SNPs with > 20% missing, > 10% heterozygosity, and < 1% minor allele frequency (Supplementary Fig. 3) to retain 10,597,683 high-quality SNPs (Supplementary Table 1). Raw indel calls underwent GATK quality filtering, and only bi-allelic variants were then retained. A resultant set of 5,717,052 indels was further filtered to obtain high-quality indels by removing indels with >20% missing calls and >10% heterozygosity and monomorphic indels and then removing indels with minor allele frequency (MAF) < 1%. From this analysis of the genomes of 781 accessions, a filtered set of 1,436,499, 17% of raw calls, were defined. The indels were then divided into 1,414,161 small indels and 22,338 structural variants (SV) (> 50 bp) (Supplementary Fig. 4). The utility of our high-quality genome-wide variation set as a true soybean haplotype map was assessed by evaluating the improvement in the power of

screening genetic variants known to control complex agronomic traits. Here we focus on the application of our genetic variant data to genomic regions that govern domestication and seed oil and protein traits, which are arguably the most frequently studied traits in soybean research.

Supplementary Note 2. Validation of SNPs and indels

The Williams 82 reference genome assembly was constructed from sequencing of multiple individuals that showed the differential segregation of several Kingwa introgression segments[8]. As expected, we found the least numbers of SNPs and indels from Williams 82K compared with any other accessions in this study. To validate our variant calling process, we examined genetic polymorphisms identified in Williams 82K, a variant of Williams 82, in both the sets of 30.8 million SNPs without filtration of < 0.01 MAF SNPs and 10.6 million SNPs with filtration of < 0.01 MAF SNPs (Supplementary Fig. 5). We found more than 1,000 homozygous non-reference variants from each of chromosomes 3, 7, 12, 14, and 15, which were also identified to contain heterogeneous regions by Haun *et al.* (2011) except chromosome 12. The rest of chromosomes contained low number of homozygous SNPs ranging from 9 to 226 in both the unfiltered and filtered set. Homozygous indels showed similar distribution patterns as those of homozygous SNPs, although more indels were filtered out by the 0.01 MAF filtration from homogeneous than heterogeneous chromosomes. Most of heterozygous variants also appeared to be identified from the chromosomes containing the presumed heterogeneous regions, however several hundreds of variants were occasionally identified across the homogeneous chromosomes, indicating that large portions of heterozygous variants identified in Williams 82K might reflect basal levels of potential spurious variants arising from paralogs[9]. Therefore, considering the fact that the Williams 82 reference genome assembly contained the regions of heterogeneity, the false-positive error rate of variant calling that was estimated by the percentage of polymorphic sites across the homogeneous chromosomes of the Williams 82 reference genome was < 0.01%.

To validate our 10.6 million SNP dataset, we used genotyping data from the 180K SoyaSNP array because 180K SoyaSNP array data from 685 of the resequenced 781 accessions were available[1]. We used 117,095 high-quality SNPs left after filtration of the SNP array data. Of the 117,095 SNP sites, 115,968 sites that matched with SNP sites in the 10.6 million SNP dataset were used to calculate genotype concordance rate between the two SNP datasets. Of the 685 SNP array samples, 63 samples had high heterozygous SNPs

ranging from 5% to 25% and thus were excluded from further comparison. Median concordance rate from the 622 samples was 97.40%. Median concordance rate from *G. max* samples (n = 353, median = 98.43) was higher than those from *G. soja* (n = 247, median = 96.27) and hybrid samples (n = 12, median = 96.11) (Supplementary Fig. 6). Given that the SNP array data showed approximately 1.25% inconsistency based on the analysis of redundant samples and SNP probes for the SNP array were biased for *G. max*[1], accuracy of genotypes in our 10.6 million SNP dataset is likely more than 99%, which is comparable to the accuracy achieved with the previous genome resequencing studies[9–11]. Nevertheless, we observed that 97 of the 622 samples had < 95% concordance rates. The low concordance rates in half of those samples were likely due to quality of the SNP array genotyping data because they contained > 2.5% heterozygous or > 2.5% missing rates. However, < 80% concordance rates of approximately 20 of the samples could not be explained by the quality estimates of the SNP array data. Because phenotypes of seeds and plant architecture especially for *G. max* are visually distinctive and automatic sample handling has been routinely practiced, this number is too high to be mislabeling or mishandling of plants or DNAs. Rather we speculate that, as we observed in the highly heterozygous samples above, another high possibility is outcrossing between soybean accessions and subsequent homogenization from several times of selfing because we have resequenced most of accessions 3 or 4 years after the SNP array genotyping study. Indeed, all of approximately a hundred of samples that both SNP array genotyping and genome resequencing were conducted at the same or next year showed > 95% concordance rates. Therefore, this study suggested that unlike conventional practice, soybean researchers may have to pay extra attention to purity of soybean accessions during germplasm maintenance.

Supplementary Note 3. Population structure

Principal component analysis **(**PCA)[12] and fastSTRUCTURE[13] were used to infer population structure of the 781 soybean set using 10.6 million SNPs (Fig. 1). The population structure of the 781 soybean set was similar to that observed in our previous analysis of 3,016 non-redundant soybean accessions genotyped using the 180K SoyaSNP array[1]. As observed in the previous study, the 781 accessions were clearly divided into one *G. max* group and two *G. soja* groups (Supplementary Fig. 7) with a distinct subgroup of *G. soja* accessions collected from the middle region of the Yellow River basin. Both scree plot from the PCA and the estimated marginal likelihood plot from the fastSTRUCTURE that showed steep slopes up to

K = 3 supported the presence of three distinct groups (Supplementary Fig. 8), although the slopes did not level off likely due to subgroupings within the large groups. While the majority of South Korean domesticated accessions formed a dense subgrouping due to recent overcollection, *G. max* accessions in general did not show distinct subgrouping on a geographic basis. Relative to landraces, the improved cultivars also appeared to be narrowly clustered within the landrace group. The groupings of wild soybean accessions were consistent with the geographic distributions of the collection sites, although several Korean accessions were grouped with a small number of Japanese accessions unlike the previous study. A wild accession from Taiwan was grouped together with Chinese accessions. Two hybrid accessions that had slightly higher than 30% *G. max* genomic fraction at *K* = 2, which was our threshold for the designation of admixture, appeared at the margin of the wild soybean group in the PCA plot. Thus, by defining a hybrid subpopulation, we show two distinctly separated soybean subpopulations, which are *G. max* and *G. soja*.

The population relationship inferred from Neighbor joining tree analysis is consistent with results obtained from our PCA and admixture analyses (Fig. 1 of the main text). The tree showed that, relative to *G. soja* accessions, *G. max* accessions formed a monophyletic cluster. Hybrid accessions appeared to cluster between the two large groups, while some accessions that have disproportionately high portions of *G. soja* or *G. max* genomic fractions clustered within *G. soja* or *G. max* groups, respectively. The tree topology is also consistent with those obtained using the 180K SNP array data except the terminal branch lengths[1]. The branch lengths of wild soybean accessions tended to be much longer than those of *G. max*, confirming that similar branch lengths between *G. max* and *G. soja* in the recent analysis of SNP array data were due to ascertainment bias that more SNPs were selected from *G. max* than from *G. soja*[14].

Supplementary Note 4. Comparison between selective sweeps and locations of cloned genes in soybean

Although most of the major, canonical domestication genes have been cloned in grass crop species including maize and rice[15], only four genes have been cloned with implication of domestication selection in soybean[16–19]. Among the four, the chromosomal location of only one gene, *Bloom1*, corresponded with a XP-CLR peak. The other three genes, *GmHs1-1*, *SHAT1-5*, and *GmTfl1*, did not overlap with XP-CLR peaks but were located right next to those peaks (Fig. 3). Swarm *et al.* (2019) speculated that *SHAT1-5*, which was cloned on the

basis of comparative homologue analyses to *Arabidopsis* genes, may not be a canonical domestication gene because of no detection of QTL in their population and, instead, *Pdh1* cloned from a cross between domesticated accessions[21] is likely a domestication gene. However, we did not detect any overlapping between these two genes and our XP-CLR peaks.

Although XP-CLR is based on multilocus allele frequency differentiation between two populations to detect selective sweeps, gene(s) under selection, domestication gene(s) in this study, at the identified selective sweeps would show striking allele frequency difference between the two populations. We observed that one major allele (45,379,743 on chromosome 2) of *GmHs1-1* and two major alleles (35,163,354 and 35,164,070 on chromosome 13) of *Bloom1* in the *G. max* population appeared to be minor alleles in the *G. soja* population, indicating that haplotypes containing these nonsynonymous mutations have been under selection during the domestication process (Supplementary Data 2). Allele frequencies (0.026, 0.021, and 0.149, respectively) of the three alleles were very low but much higher than 0.1% that is a threshold for rare alleles in human genetics[22] and they are also segregating in domesticated soybean population. *Bloom1* located on the identified selective sweeps, while *GmHs1-1* located on a windows with XP-CLR score above upper quantile score (27.1) of the XP-CLR score distribution. Collectively, our results suggested that *GmHs1-1* and *Bloom1* are weak domestication genes or hitchhikers. However, allele frequency comparison suggested that none of the nonsynonymous SNPs detected at the CDS regions of the other three genes, *SHAT1-5*, *Pdh1*, and *GmTfl1*, appeared to be under selection. It was reported that *SHAT1-5* contains causal mutations at its promoter region[17] and this gene located in a windows with XP-CLR score above upper quantile score of the XP-CLR score distribution. *Pdh1* and *GmTfl1* are located in windows with XP-CLR scores close to lower quantile score (6.4) of the XP-CLR score distribution. A determinate allele of *GmTfl1* appeared to be a novel mutation because this allele with allele frequency of 0.643 is absent in the wild population. Therefore, our results suggest that these three genes have not been under selection and should be regarded as improvement genes.

Supplementary Note 5. Candidate domestication genes

The selected mutations during domestication can be novel or standing genetic variation[23]. Novel domestication alleles such as those for the reduction of seed-shattering may be deleterious in the wild and would be rare in the wild plants. Standing domestication alleles

such as those for seed size may be fixed in domesticated plants but are segregating in the wild. Thus, standing domestication alleles may be indistinguishable from hitchhiking genes in selective sweeps defined by the XP-CLR scan. However, deleterious alleles that are major or fixed in domesticated population but are rare in wild progenitor population might be the target of selection. The comparison between identified selective sweeps and domestication QTL supported their high correlation. We observed that two of the cloned soybean domestication-implicated genes are weak domestication genes or hitchhikers. The genetic load analysis in selective sweeps corroborated unique biological feature of soybean in domestication. Therefore, we searched for nonsynonymous alleles that have been almost fixed in the domesticated population (allele frequency > 0.99, > 414.5 of 418 accessions) and rare in the wild population (allele frequency < 0.01, < 3.5 of 345 accessions) and examined how many of those alleles belongs to the selective sweeps identified in this study. We found 29 such genes (in terms of SNP sites, a total of 32 SNPs because two genes contained multiple such SNPs) (Supplementary Data 3). Of these, 26 located at the selective sweeps with > 89.4 of XP-CLR scores identified in this study and the other five located within 200 kb from the identified selective sweeps. Twenty-six alleles were predicted to be tolerated with SIFT scores > 0.05. Interestingly, 21 alleles had GERP scores > 2, which is our cut-off score for deleterious alleles and 24 alleles had derived reference allele status. Together with the difficulty of the correction of reference bias, the results suggested that GERP scores were more reliable than SIFT scores for assignment of deleterious alleles. Therefore, the estimation of GERP-based mutation load is likely more reliable than that of SIFT-based. None of the 29 genes are homologues of cloned canonical domestication genes and have been characterized with implication for domestication, suggesting that they are likely to be specific to soybean or eudicot crop plants.

Supplementary Note 6. Re-analysis of existing GWAS with SoySNP50K array genotyping and phenotyping data for oil and protein contents

A recent study[24] reported an extensive protein and oil GWAS that analyzed the accumulated historical phenotypic data from USDA GRIN database (https://www.ars-grin.gov/) and the SoySNP50K data[25] from 12,116 soybean accessions. We re-analyzed the previous GWAS because the reference genome version was updated from Glyma1 to Wm82.a2.v1 and because we noticed that the 12,116 soybean accession set contained many nearly identical accessions. With a cut-off threshold of IBD > 0.98, we removed 3,272 of nearly identical

accessions. We then performed PCA on the resultant 8,844 accessions. Scree plot from principal component (PC) analysis of the filtered 8,844 set steeply decreased up to PC number = 3 and did not level off up to PC number = 5 (Supplementary Fig. 14). The results are consistent with previous studies that large *G. max* germplasm collections were divided into three large subpopulations and two additional small subpopulations[1,24], indicating that updating and subsequent further filtration of markers and removal of redundant accessions did not change the structure of the original population.

To test whether the removal of redundant accessions improved GWAS resolution, we performed GWAS in both the 12,116 and the 8,844 accession sets using both univariate LMM and multivariate LMM (mvLMM) models. GWAS resulted in similar significant associations between both the sets in general (Supplementary Fig. 15). However, $-\log_{10}(P)$ values of the most significant SNPs in major peaks are higher in the original 12,116 set than the 8,844 set and more minor significant peaks appeared in the original 12,116 set relative to the 8,844 set. The results indicated that filtering redundant samples improved the resolution of our GWAS likely due to reducing the exaggeration of association signals from repeated use of nearly duplicated samples[26].

Our GWAS results on the 8,844 accessions were quite similar to the previously reported[24] results on the 12,116 accessions with two notable exceptions. First, a minor peak on chromosome 8 for oil in the previous study, whose existence was not mentioned in the report likely because of being too minor, appeared to be a clearly visible major peak in our 8,844 set GWAS. Second, we observed a novel peak at 21 Mb on chromosome 20. We think that these novel peaks likely appeared due to the update of the soybean reference genome sequence. Notably, a sequence contig (~ 3.4 kb) containing the peak SNP (BARC_1.01_Gm_20_30930931_A_G) was displaced from the well-known major peak position at 30 Mb to the 21 Mb position during the version update from the soybean reference genome Glyma1 to Ws82.a2.v1 (Supplementary Table 3). Because no significant linkage or association signal for oil or protein contents has been reported in this region so far, we think that the additional major peak at chromosome 20 is likely to be an artifact related to the assembly problem of the soybean reference genome Ws82.a2.v1. Thus, in subsequent analyses, we did not pay attention to this peak. Interestingly, the major peak on chromosome 8 is likely true positive because this peak was recently detected as a major QTL for oil content from an interspecific soybean mapping population[27].

It is well known that seed protein content has a negative relationship with seed oil content in soybean[28], and Bandillo *et al.* (2015) also reported negative relationships between oil and protein association signals. Thus, we applied mvLMM model to identify genomic regions that exhibited pleiotropy for protein and oil contents. Distribution of significant SNPs from mvLMM model on the Manhattan plot looked like putting oil-LMM GWAS on protein-LMM GWAS. Among five major peaks, peaks on chromosomes 5 and 8 appear to be oil-specific peaks, a peak on chromosome 13 to be protein-specific, and peaks on chromosome 15 and 20 to be pleiotropic peaks. It should be noted that trait-specific peaks on chromosome 8 and 13 are novel peaks, which were missing in a recently reported mvLMM-GWAS analysis for seed oil and protein[29]. Because the mvLMM-GWAS results looked like the overlapping of the two univariate LMM-GWAS results and our objective for GWAS is to examine the possibility of improving GWAS resolution from the imputation of genome resequencing panel data into an existing GWAS, we focused on results from mvLMM results for a comparison between the existing GWAS and imputed GWAS.

Supplementary Note 7. Imputation of existing SoySNP50K array genotyping data with genome sequencing panel data

We imputed 4,467,134 SNPs on chromosomes from 418 *G. max* accessions into 36,489 SNPs from SoySNP50K data of 8,844 soybean accessions. In the final imputed data, ~16% (5,868) of SNPs from the SoySNP50K data were lost. The lost 5,868 SNPs from the SNP array are those that are not overlapping with positions in the set of the 4.5 million SNPs. From the 62.9 million raw SNPs, we found 5,130 of the 5,868 SNP positions, however 1,634 positions (31.9%) of 5,130 were overlapping deletions and 1,305 (25.4%) of them were multi-allelic sites. The results suggested that probes of the lost 5,868 SNPs were likely designed from highly variable, low-quality SNP loci that were called from low-depth resequencing data obtained from eight soybean genotypes[30]. To evaluate imputation accuracy of the imputed SNPs, we examined the distribution of accuracy (squared correlation r^2) values across MAF spectrum (Supplementary Fig. 16). We observed that at the low end of the MAF spectrum (MAF < 0.1), median accuracy values were < 0.5 with wide value distributions. At most of the MAF bins that were > 0.1 MAF, median accuracy values were higher than 0.8 and their distributions were narrower than those at the low end of the MAF spectrum. Imputation with Beagle uses linkage disequilibrium structure in a chromosomal region to infer the alleles of SNPs. Thus, we investigated a relationship between linkage disequilibrium structure and

imputation accuracy by comparing the distribution patterns of the accuracy values across the MAF spectrum between euchromatic and heterochromatic regions, which showed approximately a fourteen-fold difference in the LD decay distance (Supplementary Fig. 9). Both median accuracy values and accuracy distributions at the heterochromatic regions were much higher and narrower than those at the euchromatic regions. Although SNP density (55.8 SNPs/Mb) across the euchromatic regions of the SoySNP50K array data used in this study is approximately five-fold higher than that (11.5 SNPs/Mb) across the heterochromatic regions (Supplementary Fig. 16), our results suggested that the different imputation performance might be due to that the euchromatic regions actually contained sparser marker density relative to that of the heterochromatic regions in relation to LD distance. Finally, the imputed set of 4,461,266 SNPs underwent further filtration to remove poorly imputed (Beagle $r^2 < 0.3$) and low MAF (< 0.01) SNPs. The resultant high-quality 3,082,234 SNPs with median Beagle r^2 of 0.95 were used for subsequent GWAS.

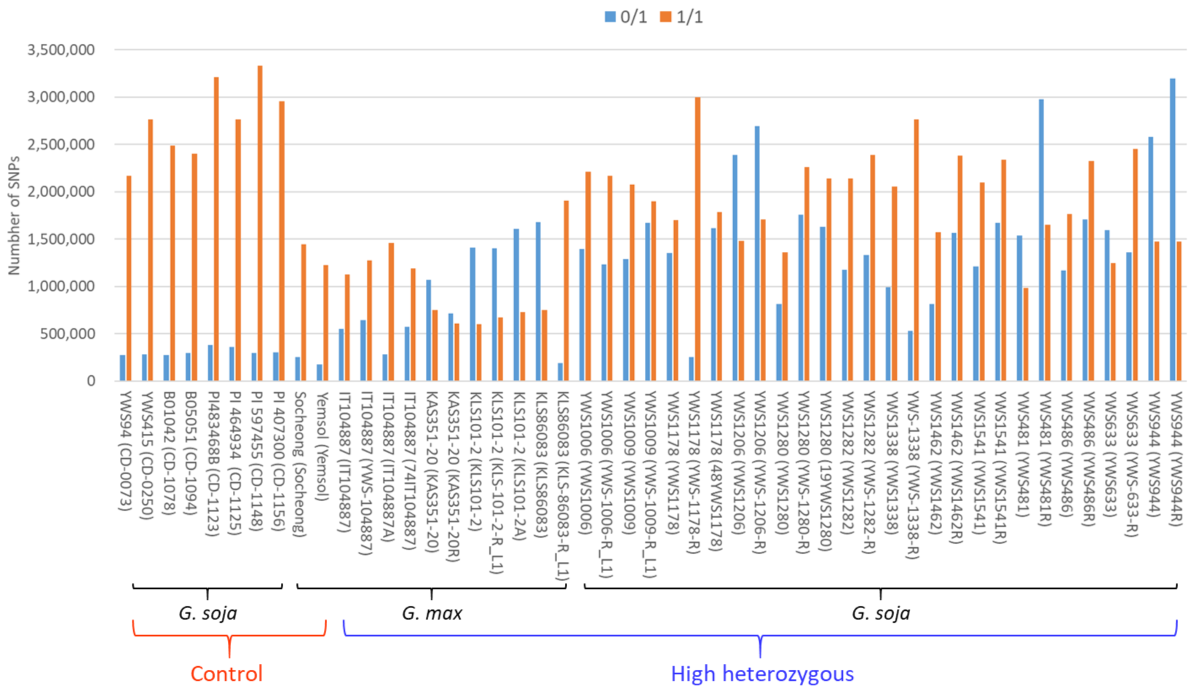

Supplementary Figure 1. Distribution of heterozygous and homozygous SNP genotypes called from a set of soybean samples consisting of 27 soybean accessions and 49 samples. Number of raw SNPs obtained from the GenotypeGVCF step of the Genome Analysis Toolkit (GATK) Best Practices are shown. Reference allele is indicated by 0, non-reference alleles is 1. Heterozygous SNPs (blue bars) and homozygous SNPs (red bars) are 0/1 and 1/1, respectively. Memberships to domesticated (*Glycine max*) and wild (*Glycine soja*) soybean are indicated under soybean accession and sample (in parenthesis) names. Controls are those samples that showed low heterozygosity in the initial sequenced samples and high heterozygous are those samples that showed higher than 0.5 million heterozygous SNPS in the first analyzed samples shown leftmost of the list of each of soybean accessions. Genome resequencing of all 17 high heterozygous samples were repeated using new libraries prepared from filial bulks of the first sequenced samples. In case of IT104887, additional resequencing data were obtained from a filial single plant (IT104887A) and using the same DNA (repeated sample, 74IT104887) as the first sample. In case of KLS101-2, additional resequencing data were obtained from a filial single plant (KLS101-2A). In cases of YWS1178 and YWS1280, additional resequencing data were obtained using the same DNAs (48YWS1178 and 19YWS1280, respectively) as the first samples.

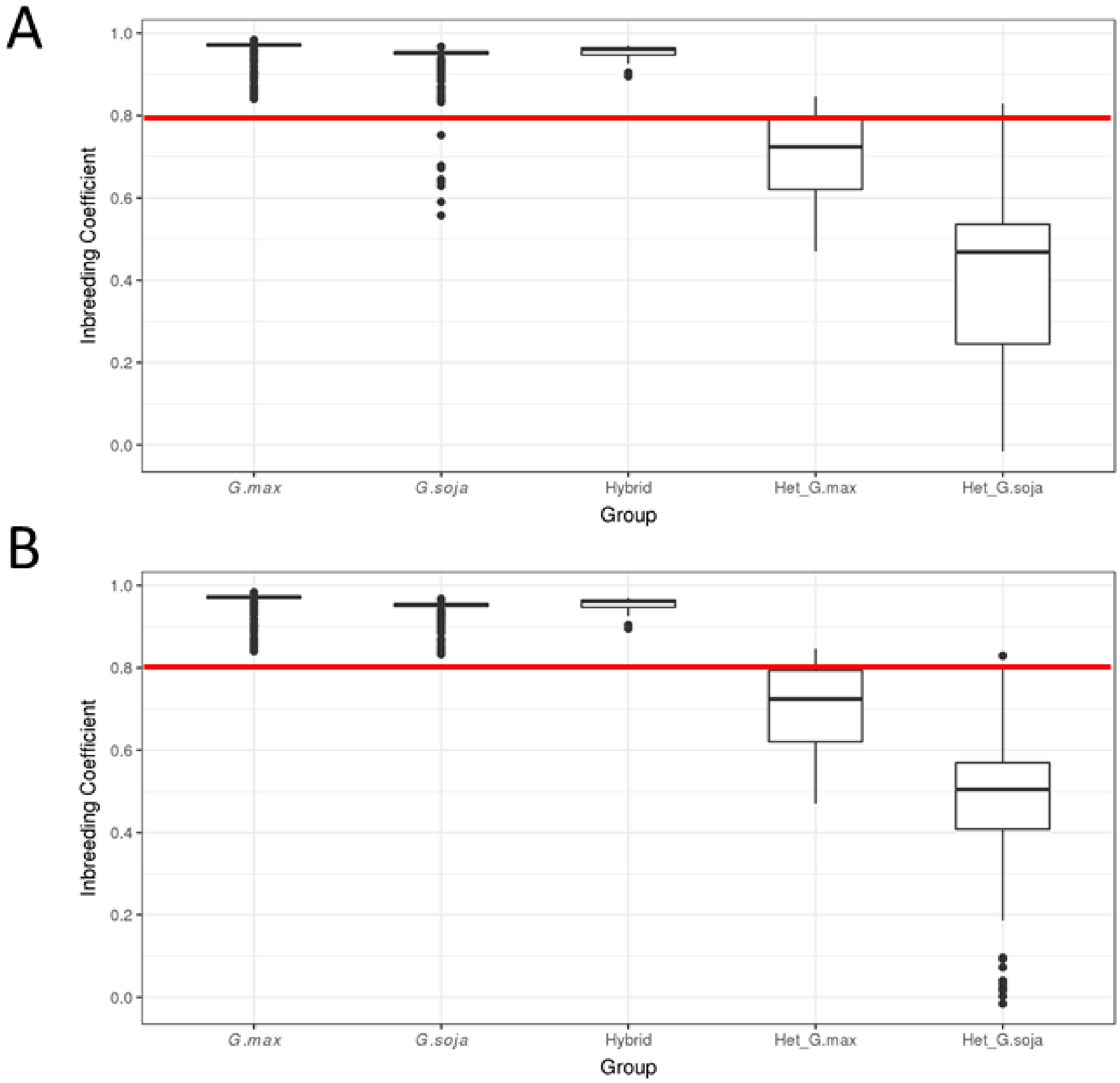

Supplementary Figure 2. Distribution of inbreeding coefficients within subpopulations of a soybean population of 855 samples. Each box represents the median and interquartile range (IQR). The whiskers represent the range of 1.5 times IQR and the dots beyond the whiskers are outlier values. Subpopulations: *G. max*, domesticated; *G. soja*, wild; Hybrid, *G. max/G. soja*; Het_G.max, high heterozygous domesticated; and Het_G.soja, high heterozygous wild soybean subpopulation. (**A)** Distribution of individual inbreeding coefficients in subpopulations (*G. max, n* = 418; *G. soja, n* = 353, Hyhrid, *n* = 18, Het_G.max, *n* = 22; and Het_G.soja, *n* = 44). (**B)** Distribution of individual inbreeding coefficients in subpopulations (*G. max, n* = 418; *G. soja, n* = 345, Hyhrid, *n* = 18, Het_G.max, *n* = 22; and Het_G.soja, *n* = 52) after transferring eight wild accessions with inbreeding coefficient per individual of less than 0.8 from *G. soja* to high heterozygous *G. soja* subpopulation. Source data are provided as a Source Data file.

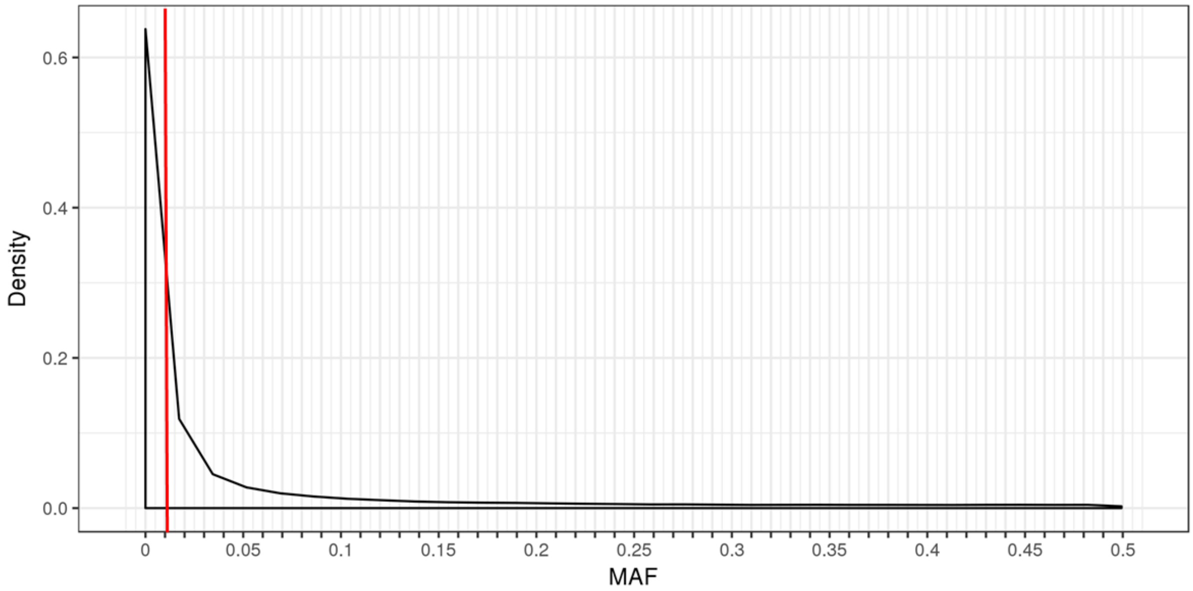

Supplementary Figure 3. Distribution of minor allele frequency (MAF) for SNPs. A red line indicates 1% MAF.

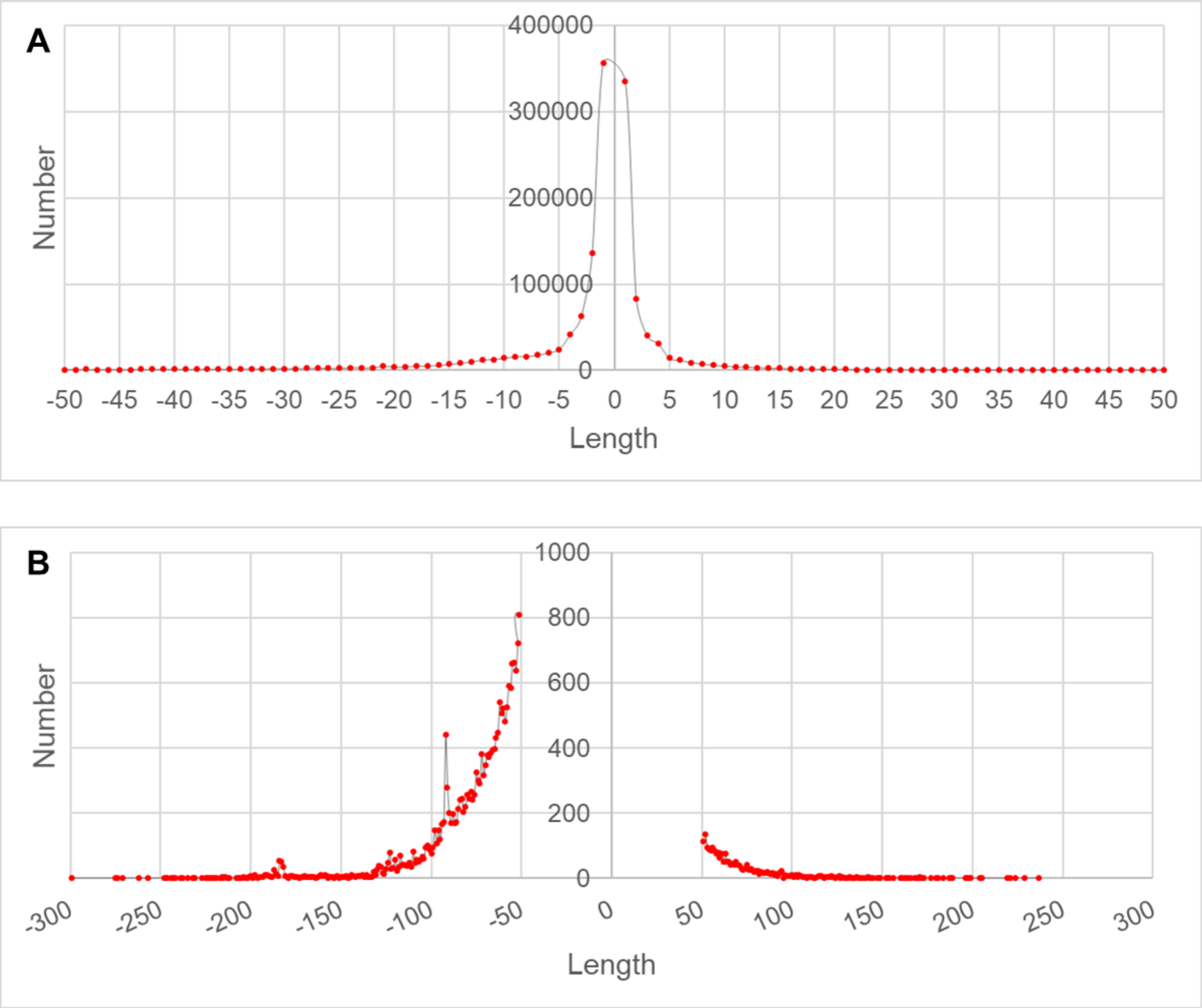

Supplementary Figure 4. Distribution of sizes of insertion/deletion (indel) variants and structural variants (SV). (**A**) Indel size distribution. (**B**) SV size distribution.

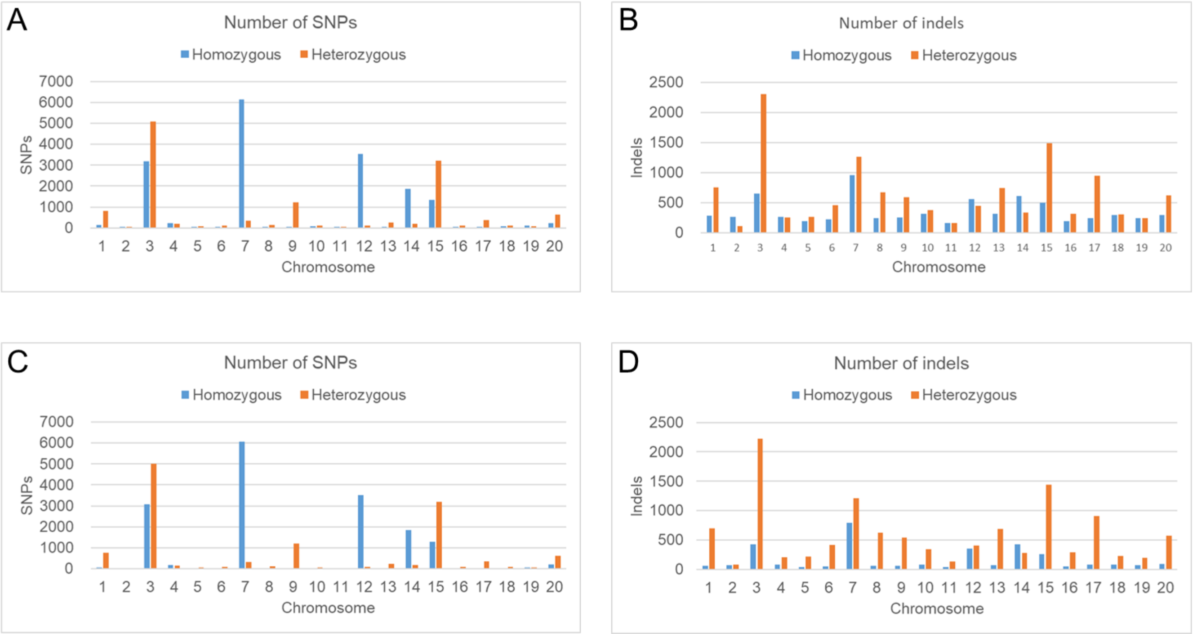

Supplementary Figure 5. Genomic distributions of SNPs and indels identified in Williams 82K. (**A**) Number of SNPs per chromosome of 17,384 homozygous non-reference and 13,237 heterozygous SNPs identified in Williams 82K in the set of 30.8 million SNPs without filtration of < 0.01 minor allele frequency SNPs. (**B**) Number of small indels per chromosome of 7,099 homozygous non-reference and 12,678 heterozygous SNPs identified in Williams 82K in the set of 5.7 million indels without filtration of < 0.01 minor allele frequency indels. (**C**) Number of SNPs per chromosome of 16,452 homozygous non-reference and 12,754 heterozygous SNPs identified in Williams 82K in the set of 10.6 million SNPs with filtration of < 0.01 minor allele frequency SNPs. (**D**) Number of small indels per chromosome of 3,276 homozygous non-reference and 11,860 heterozygous indels identified in Williams 82K in the set of 1.4 million indels with filtration of < 0.01 minor allele frequency SNPs. The numbers of homozygous SNPs and indels were greater in chromosomes 3, 7, 12, 14, and 15 than in the other chromosomes.

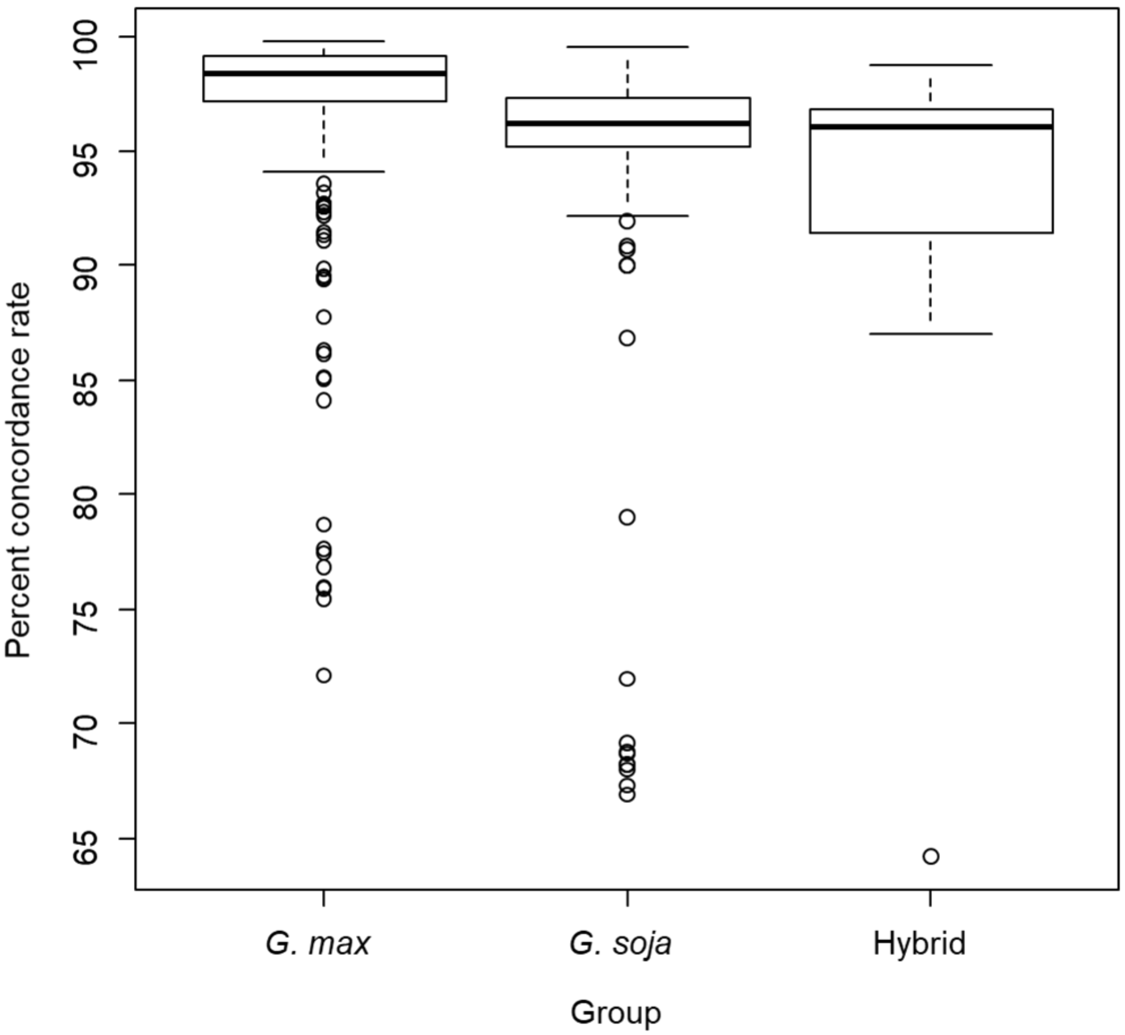

Supplementary Figure 6. Genotype concordance rates between soybean genome resequencing SNPs and 180K SoyaSNP array. Box-and-whisker plots were used to compare the median concordance rate among *Glycine max* (*n* = 353), *Glycine soja* (*n* = 247) and hybrid (*n* = 12) samples. Each box represents the median and interquartile range (IQR). The whiskers represent the range of 1.5 times IQR and the open circles beyond the whiskers are outlier values. Source data are provided as a Source Data file.

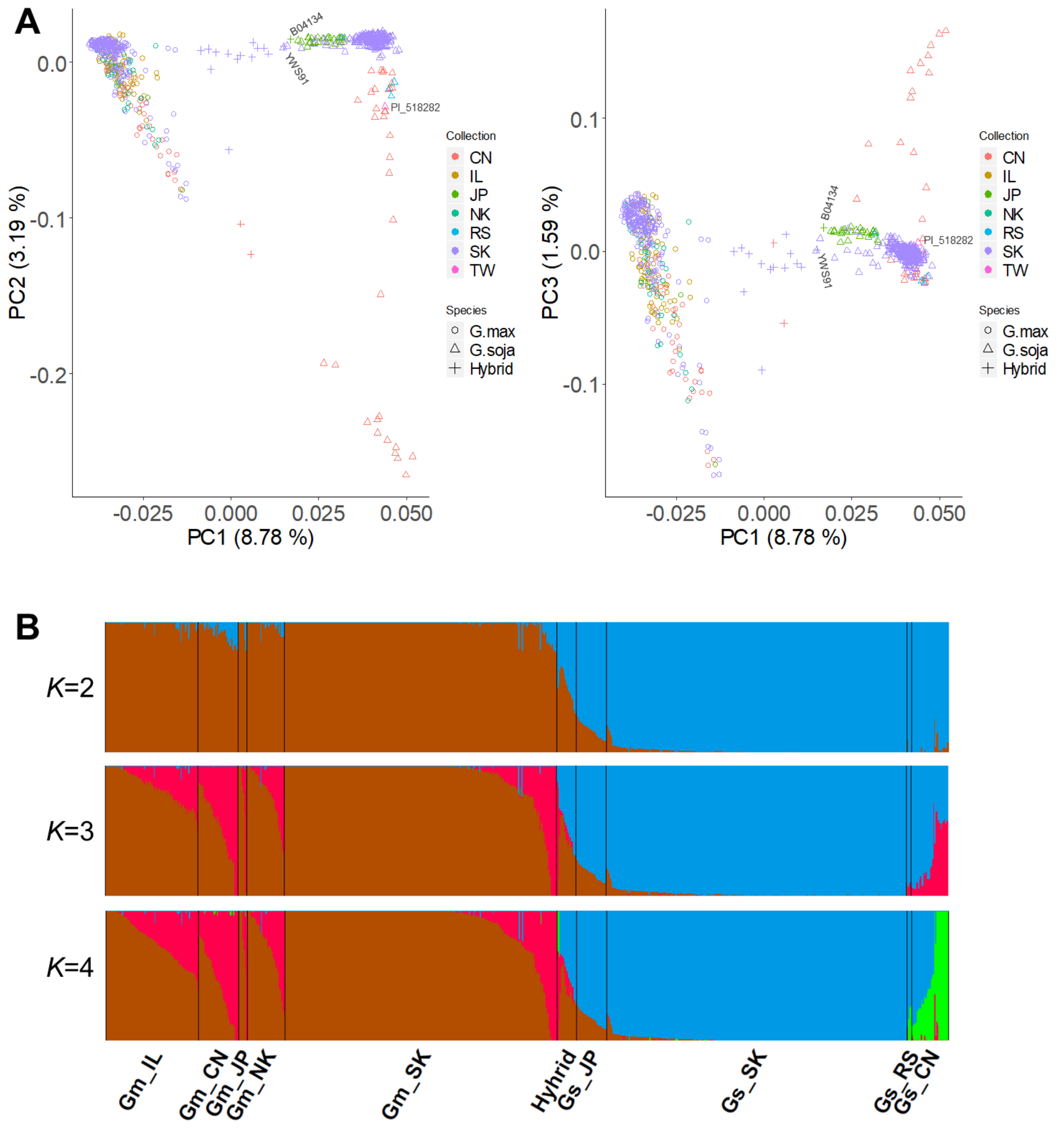

Supplementary Figure 7. Population structure of the 781 haplotype soybean accession set. (**A)** Principal components (PC) of SNP variation. The plots show the first three principal components. (**B)** fastSTRUCTURE plots. The accessions were divided into three groups: *Glycine max*, *G. soja*, and hybrids. The accessions were further indicated by the countries of collection or improvement status of the soybean accessions represented by two-letter codes—CN, China; IP, improved breeding line; JP, Japan; NK, North Korea; RS, Russia; SK, South Korea; and TW, Taiwan. Two hybrid accessions B04134 and YWS91 were located near *G. soja* accessions YWS204 and YWS1034 at the margin of a *G. soja* subpopulation. An only accession PI 549046 (*G. soja*) from Taiwan was labeled. All accessions from NK were *G. max*. In **B**, Gm and Gs are *G. max* and *G. soja*, respectively, and a *G. soja* accession from Taiwan was included in the Cs_CN group based on the PC analysis.

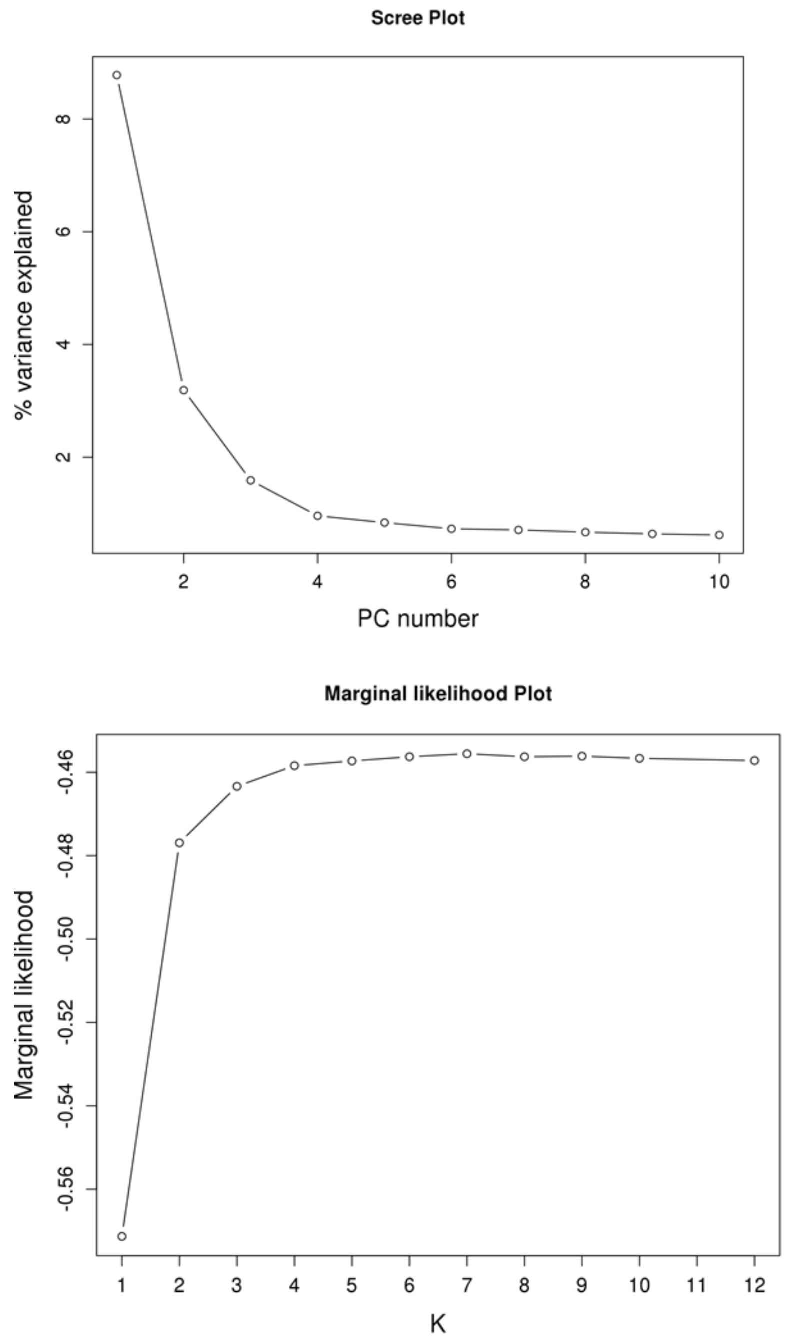

Supplementary Figure 8. Scree plot and marginal likelihood plot from population structure analyses of the 781 haplotype soybean accession. Scree plot shows the PC number and their contribution to variance from principal component analysis. Marginal likelihood plot shows the model complexity (*K*) and marginal likelihood from fastSTRUCTURE.

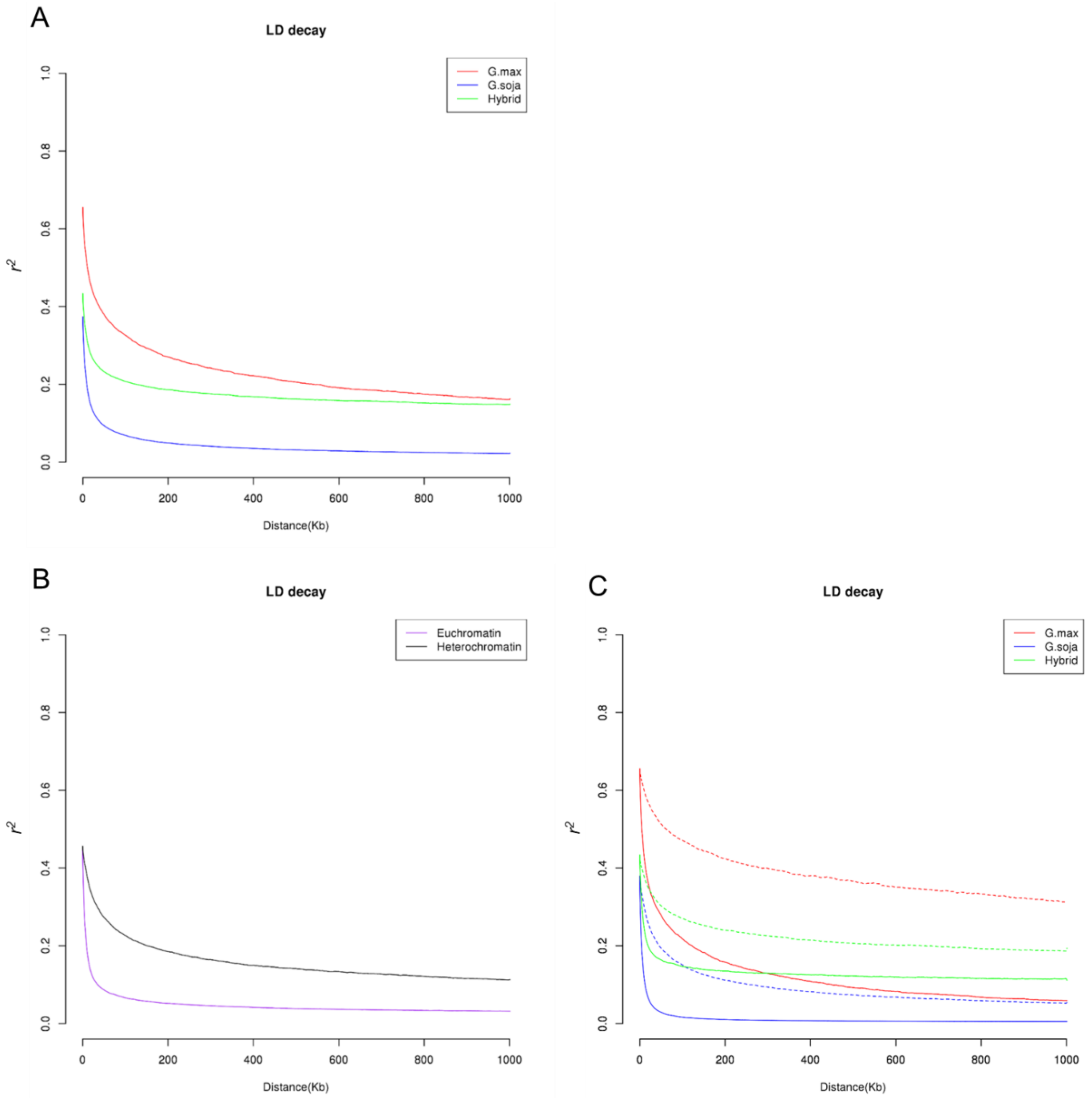

Supplementary Figure 9. Linkage disequilibrium (LD) decay in soybean genome. (**A**) Decay of LD in domesticated (*Glycine max*) and wild (*Glycine soja*) soybean and their natural hybrids. (**B**) Decay of LD in euchromatic and heterochromatic regions for total population. (**C**) Decay of LD in euchromatic (solid line) and heterochromatic (dotted line) regions for *G. max* and *G. soja* soybean and their natural hybrids. Decay rates of LD were determined by the squared correlations of allele frequencies (r^2) against physical distance (kb) between polymorphic SNP loci.

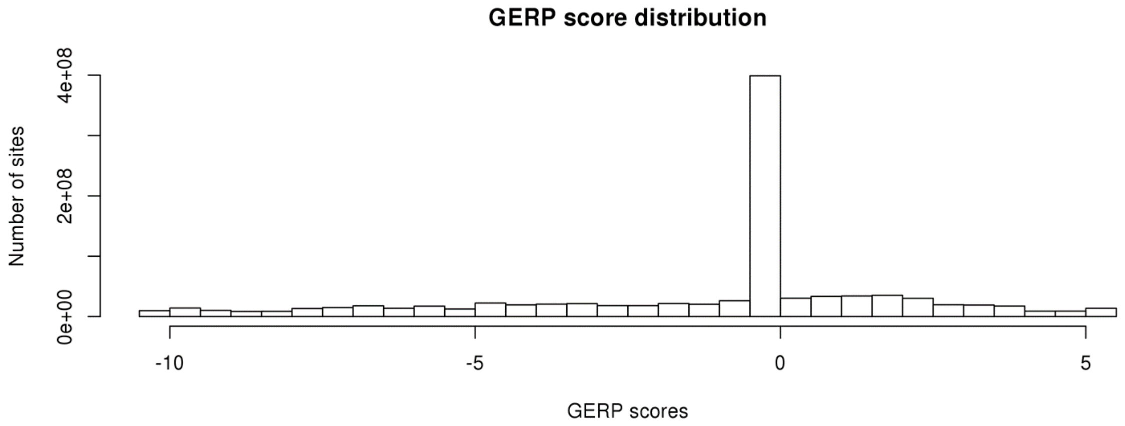

Supplementary Figure 10. Distribution of GERP scores for soybean genome.

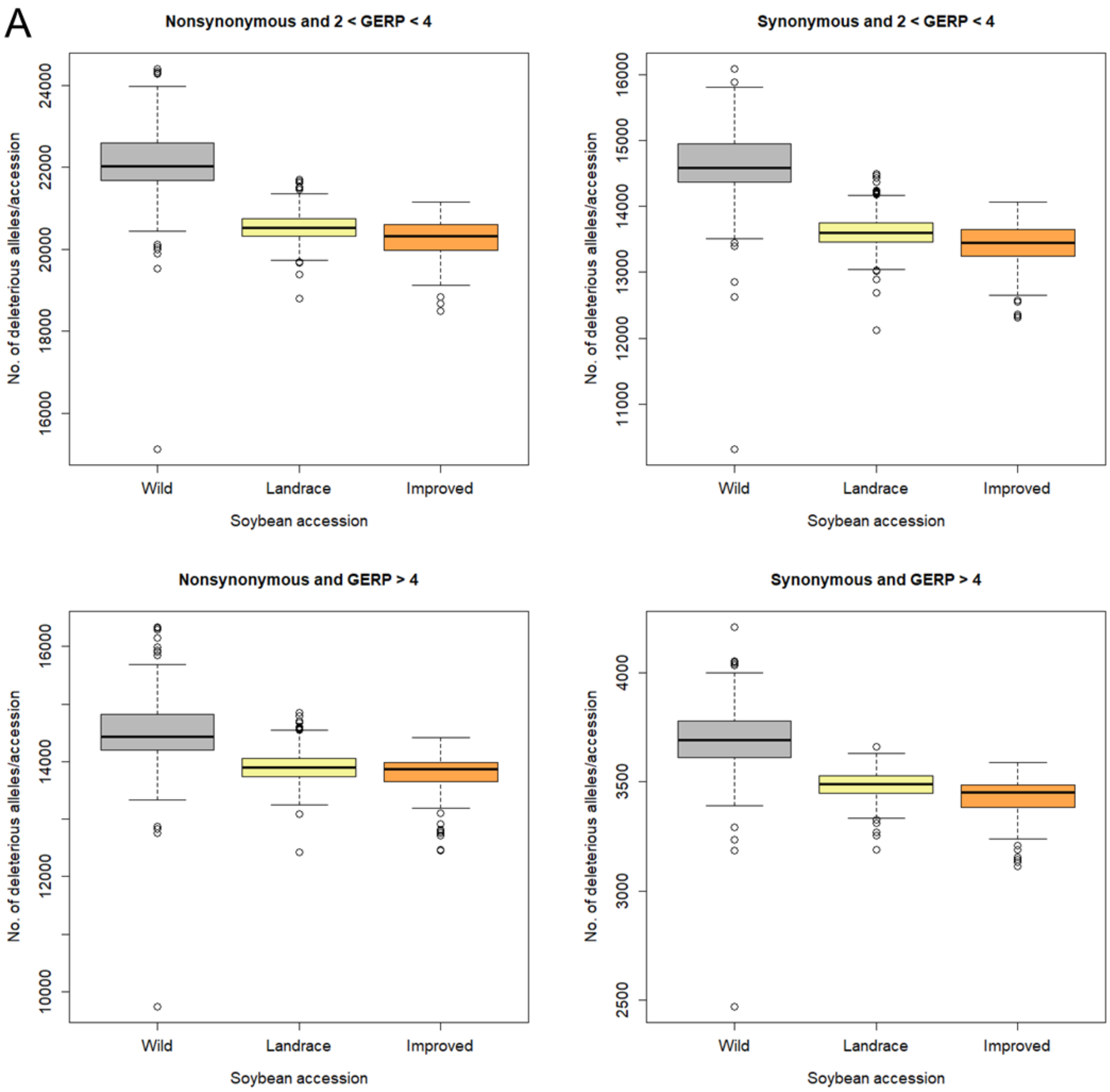

A
Nonsynonymous and 2 < GERP < 4
Synonymous and 2 < GERP < 4
Nonsynonymous and GERP > 4
Synonymous and GERP > 4
No. of deleterious alleles/accession
Soybean accession
Wild
Landrace
Improved

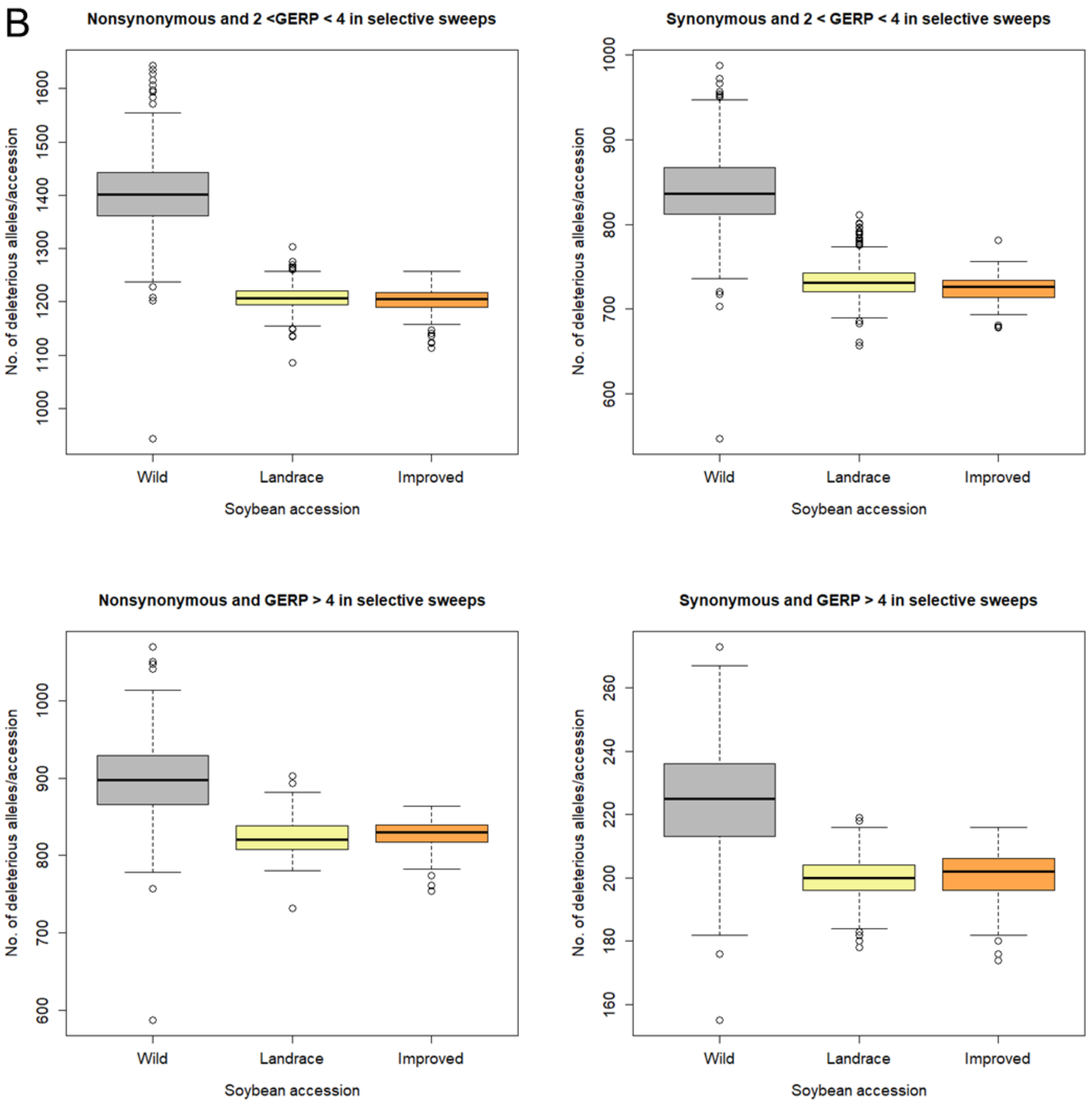

Supplementary Figure 11. Box-and-whisker plot distributions of mutation burden in domesticated (*Glycine max*, landrace cultivars = 332 and improved lines = 86) and wild (*Glycine soja*, n = 345) soybean populations. Each box represents the median and interquartile range (IQR). The whiskers represent the range of 1.5 times IQR and the open circles beyond the whiskers are outlier values. (**A**) Distribution of mutation burden per accession in each of four deleterious mutation categories is shown: nonsynonymous SNPs with 2 < GERP <4, synonymous SNPs with 2 < GERP <4, nonsynonymous SNPs with GERP > 4, and synonymous SNPs with GERP > 4. The subgroups in each of plots are significantly different between one another with $P < 0.01$ in Tukey multiple comparison tests. (**B**) Distribution of mutation burden of selective sweeps per accession in each of four deleterious mutation categories. The domesticated (landrace and improved) and wild soybean subgroups in each of plots are significantly different with $P < 1.0e\text{-}9$ in Tukey multiple comparison tests. The landrace and improved subgroups were not significantly different except the synonymous SNPs with 2 < GERP <4 ($P = 0.039$). Source data are provided as a Source Data file.

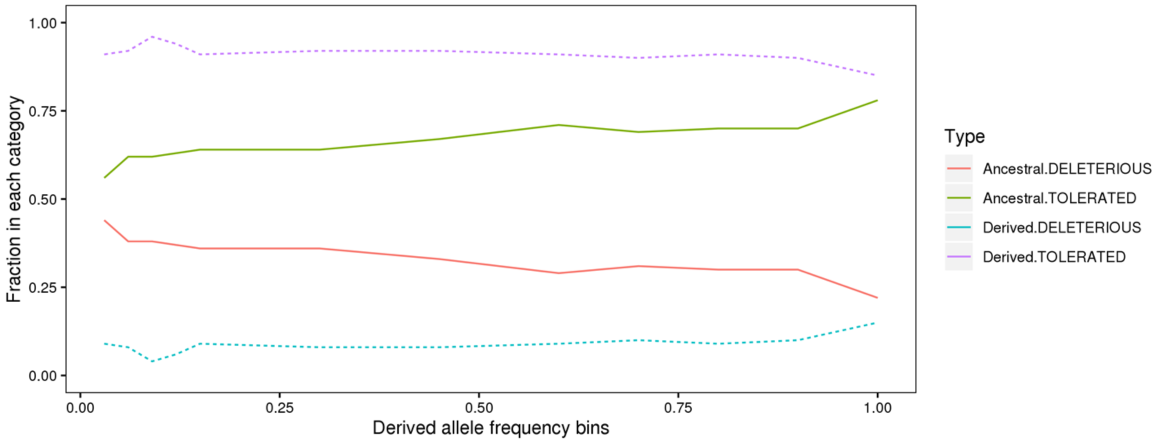

Supplementary Figure 12. Illustration of the reference bias present in SIFG 4G. The x-axis shows the mean population frequency of nonsynonymous SNPs in the 30.8 million SNP data set. The left-most bins in the x-axis cover very narrow intervals of frequencies (0.03) since most of the data are present in these bins. The y-axis plots the fraction of SNPs in each bin that are classified into Tolerated or Deleterious by the SIFT. And the y-axis is shown separately according to whether the genome reference sequence carries the ancestral or the derived allele. When the reference carries the ancestral allele, an SNP is classified as Deleterious with a probability that ranges from nearly 44% at low frequencies to 22% at high frequencies (solid red line). In contrast, for SNPs where the reference carries the derived allele, the fraction of Deleterious alleles is near 0% at all frequencies (dotted blue line).

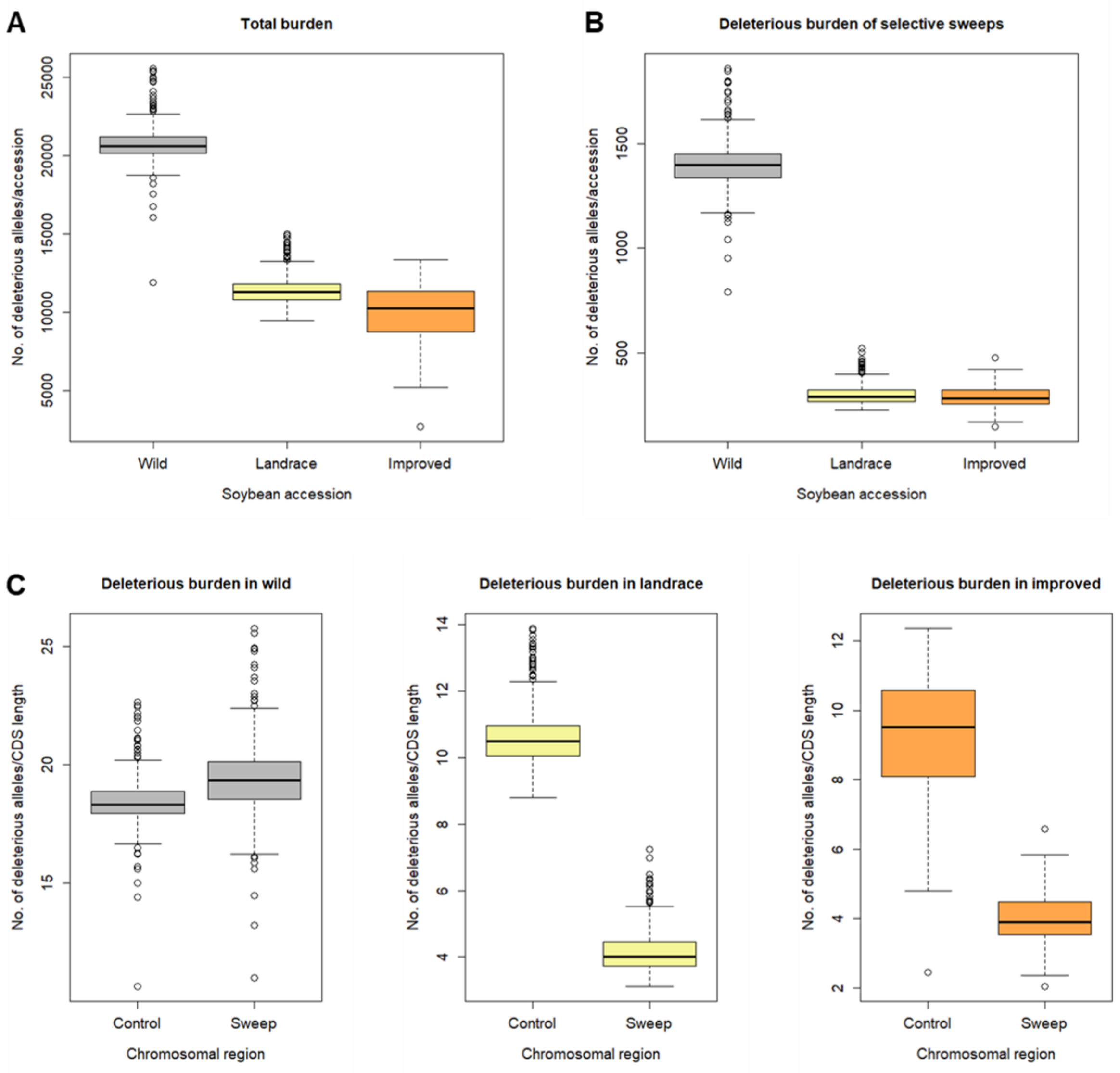

Supplementary Figure 13. Box-and-whisker plot distributions of mutation burden in domesticated and wild soybean populations estimated using nonsynonymous deleterious SNPs predicted by the SIFT analysis. Each box represents the median and interquartile range (IQR). The whiskers represent the range of 1.5 times IQR and the open circles beyond the whiskers are outlier values. (**A**) Total mutation burden in individual domesticated (*Glycine max*, landrace cultivars = 332 and improved lines = 86) and wild (*Glycine soja*, *n* = 345) soybean accessions. (**B**) Mutation burden among landrace, improved, and wild soybean accessions in domestication sweep regions. (**C**) Mutation burden in wild, landrace, and improved soybean accessions between domestication selective sweeps and control regions (rest of the genome). Vertical axis shows number of deleterious alleles per 100-kb CDS length. The subgroups in each of plots are significantly different between one another with $P < 2.2e-16$ in two-sided t-tests or Tukey multiple comparison tests except deleterious burden per accession of selective sweeps between landrace and improved soybean accessions with $P = 0.333$ at the plot B. Source data are provided as a Source Data file.

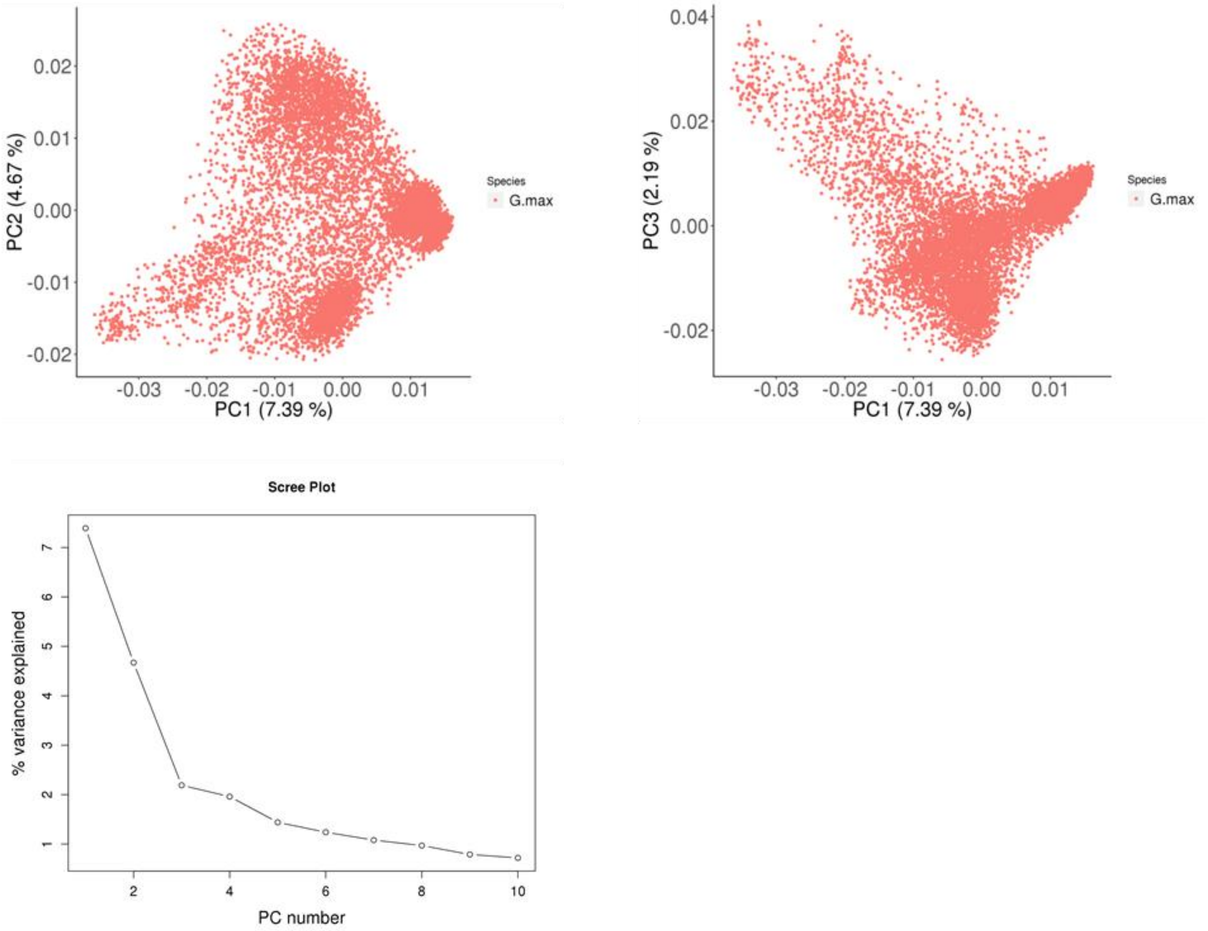

Supplementary Figure 14. Principal component analysis of 8,844 non-redundant domesticated soybean (*G. max*) accessions. The plots (top) show the first three principal components. Scree plot (bottom) of the PC number and their contribution to variance from principal component analysis.

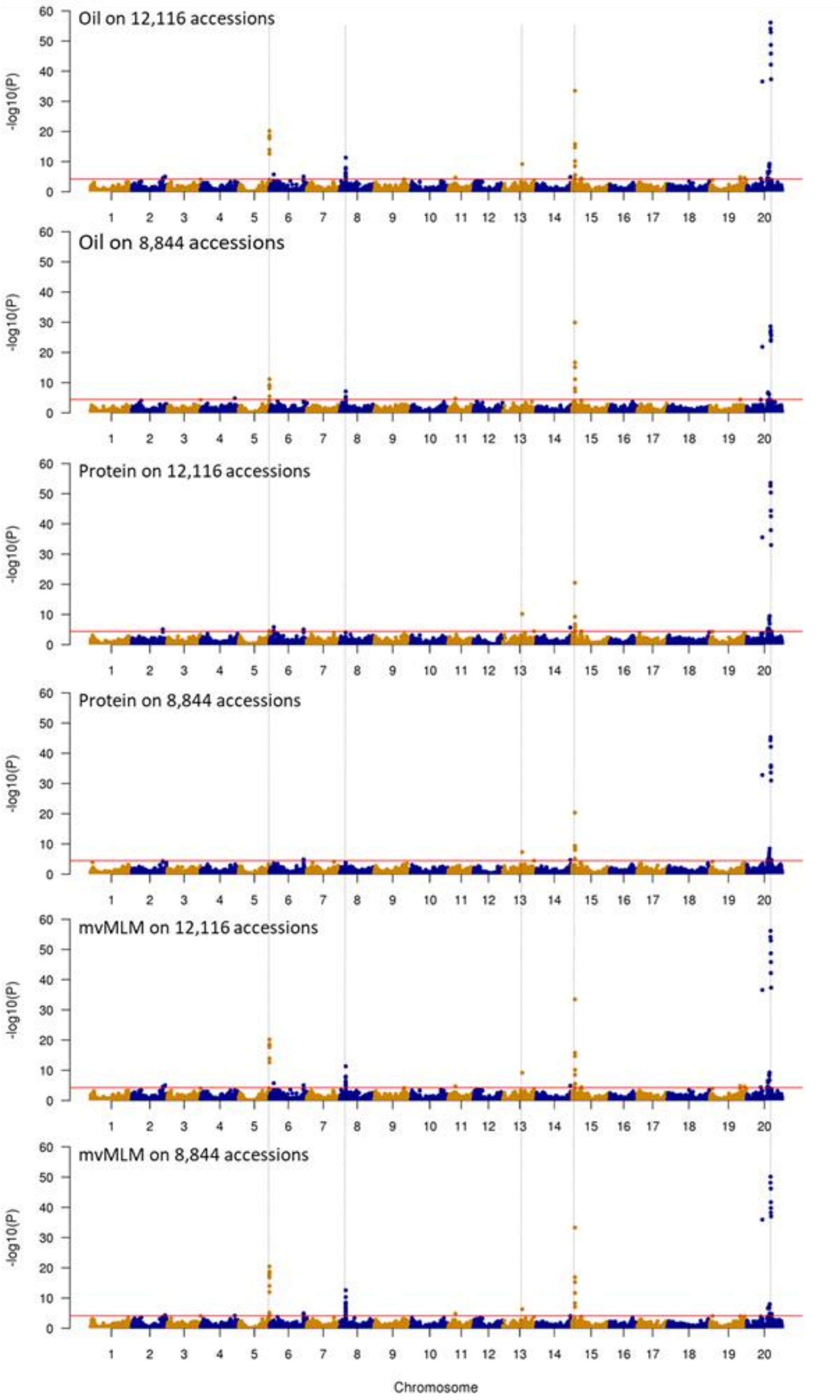

Supplementary Figure 15. Comparison of genome-wide association studies for seed oil and protein contents in both the 12,116 and 8,844 soybean accession sets using both univariate LMM and multivariate LMM (mvLMM) models. Horizontal red lines represent 5% significance thresholds corrected for multiple testing using Benjamini-Hochberg that ranged from 4.15 to 4.44. Chromosomal regions of five major peaks are indicated by dashed vertical line for comparison.

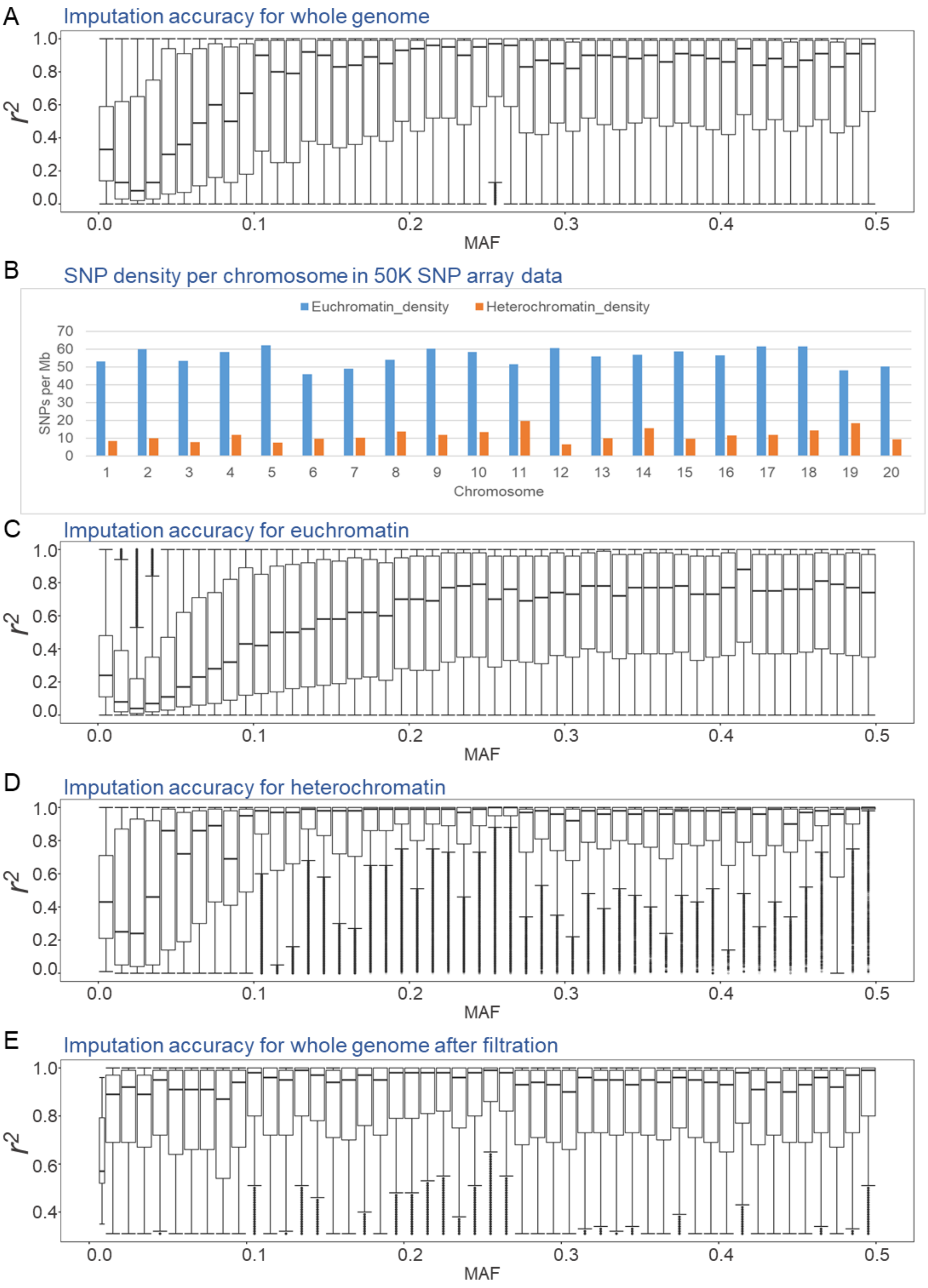

Supplementary Figure 16. Genotype imputation accuracy when imputing genotypes from 4.5 million SNPs from 418 *Glycine max* accessions into 8,844 samples genotyped with the SoySNP50K array. Imputation accuracies of imputed alleles were displayed across minor allele frequency (MAF) bins of size 0.01 for sites that were polymorphic in imputed data set. The squared correlation (r^2) between the true allele dosage and the imputed posterior

allele probability was reported for each MAF bin. Each box represents the median and interquartile range (IQR). The whiskers represent the range of 1.5 times IQR and the dots beyond the whiskers are outlier values. (**A**) Imputation accuracy values (*n* = 4,467,134) were displayed based on genome-wide MAF bins. (**B**) Distribution of relative SNP densities per chromosome between euchromatic and heterochromatic regions in the SoySNP50K data. (**C**) Imputation accuracy values (*n* = 2,443,814) were displayed based on MAF bins of SNPs in euchromatic regions. (**D**) Imputation accuracy values (*n* = 2,023,320) were displayed based on MAF bins of SNPs in hetrochromatic regions. (**E**) Imputation accuracy values (*n* = 3,082,234) were displayed based on genome-wide MAF bins after filtration of poorly imputed (r^2 < 0.3) and low MAF (0.01) SNPs. Source data are available from figshare repository (https://figshare.com/projects/Soybean_haplotype_map_project/76110).

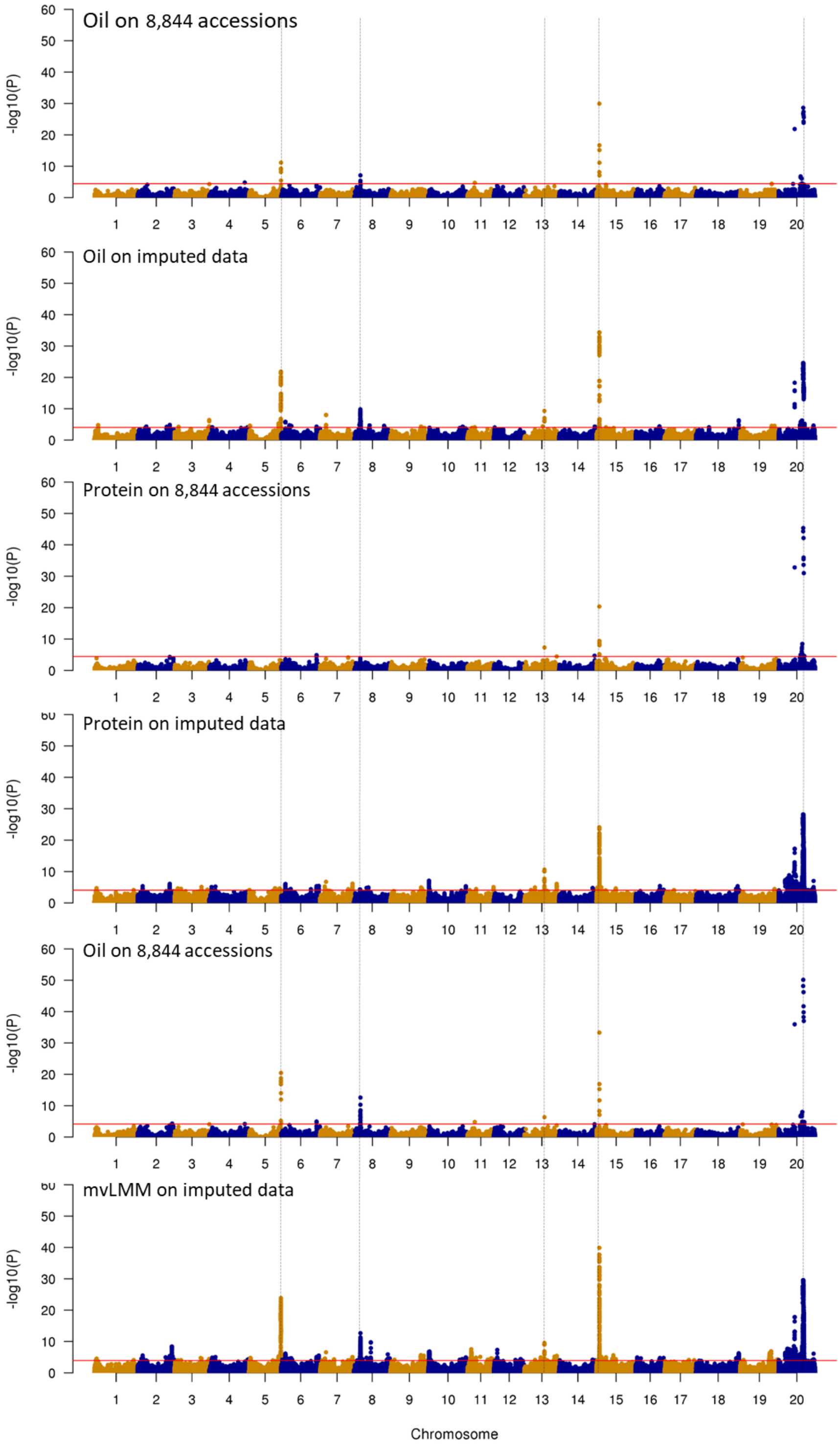

Supplementary Figure 17. Comparison of genome-wide association scans for variants associated with seed oil and protein using SoySNP50K and imputed genotype data in soybean. Six different trait-based and model-based Manhattan plots represent -log 10 (p-value) for SNPs distributed across all 20 chromosomes of soybean. Y-axis:-log 10 (p-value) and x-axis: soybean chromosomes. Horizontal red lines represent 5% significance thresholds corrected for multiple testing using Benjamini-Hochberg that ranged from 3.93 to 4.44. Univariate linear mixed model and multivariate linear mixed model (mvLMM) were used.

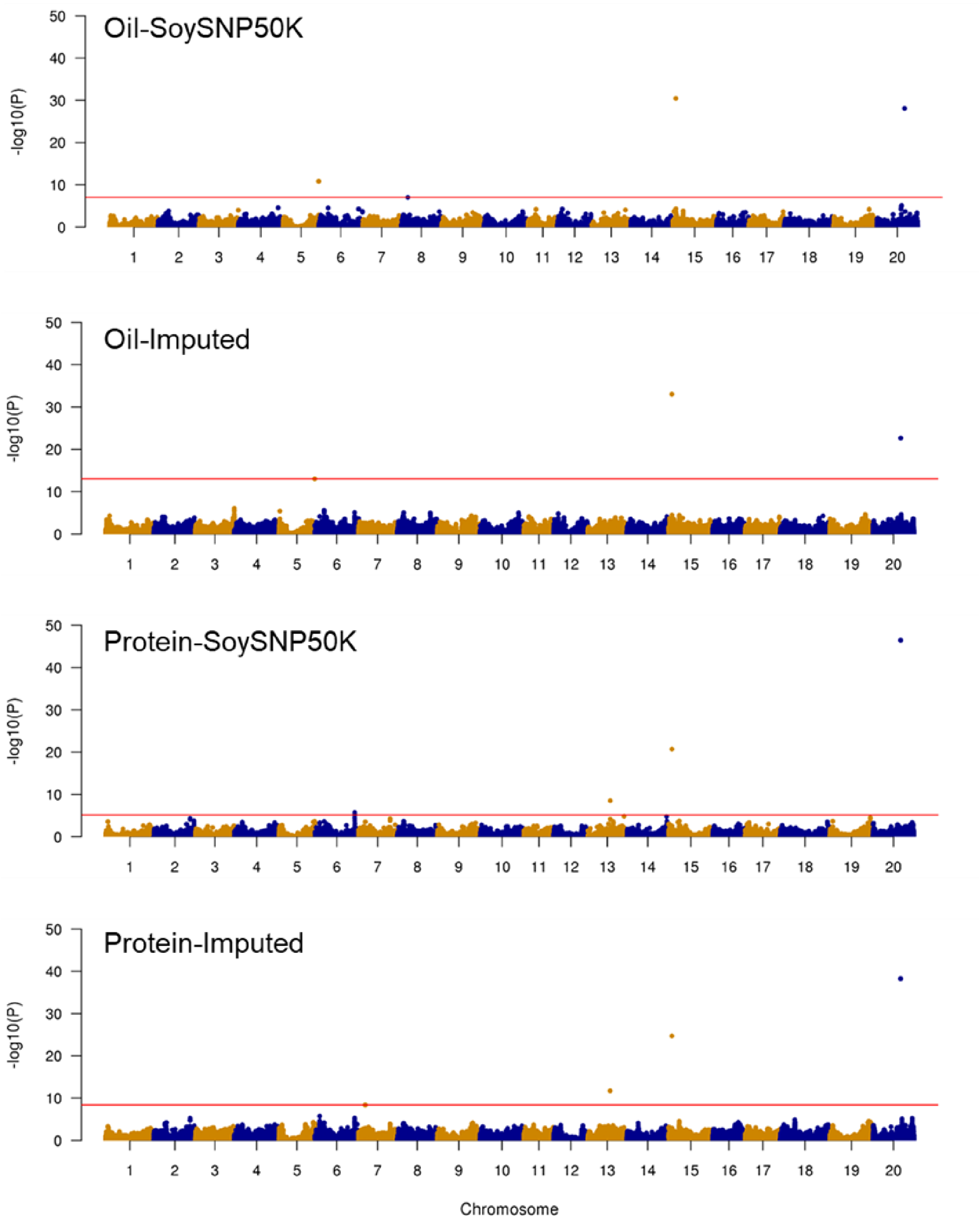

Supplementary Figure 18. Comparison of genome-wide association studies using multi-locus mixed model (MLMM) for variants associated with oil and protein using SoySNP50K and imputed genotype data in 8,844 soybean accessions. Horizontal red lines represent 5% significance thresholds corrected for multiple testing using Benjamini-Hochberg that ranged from 5.17 to 13.2.

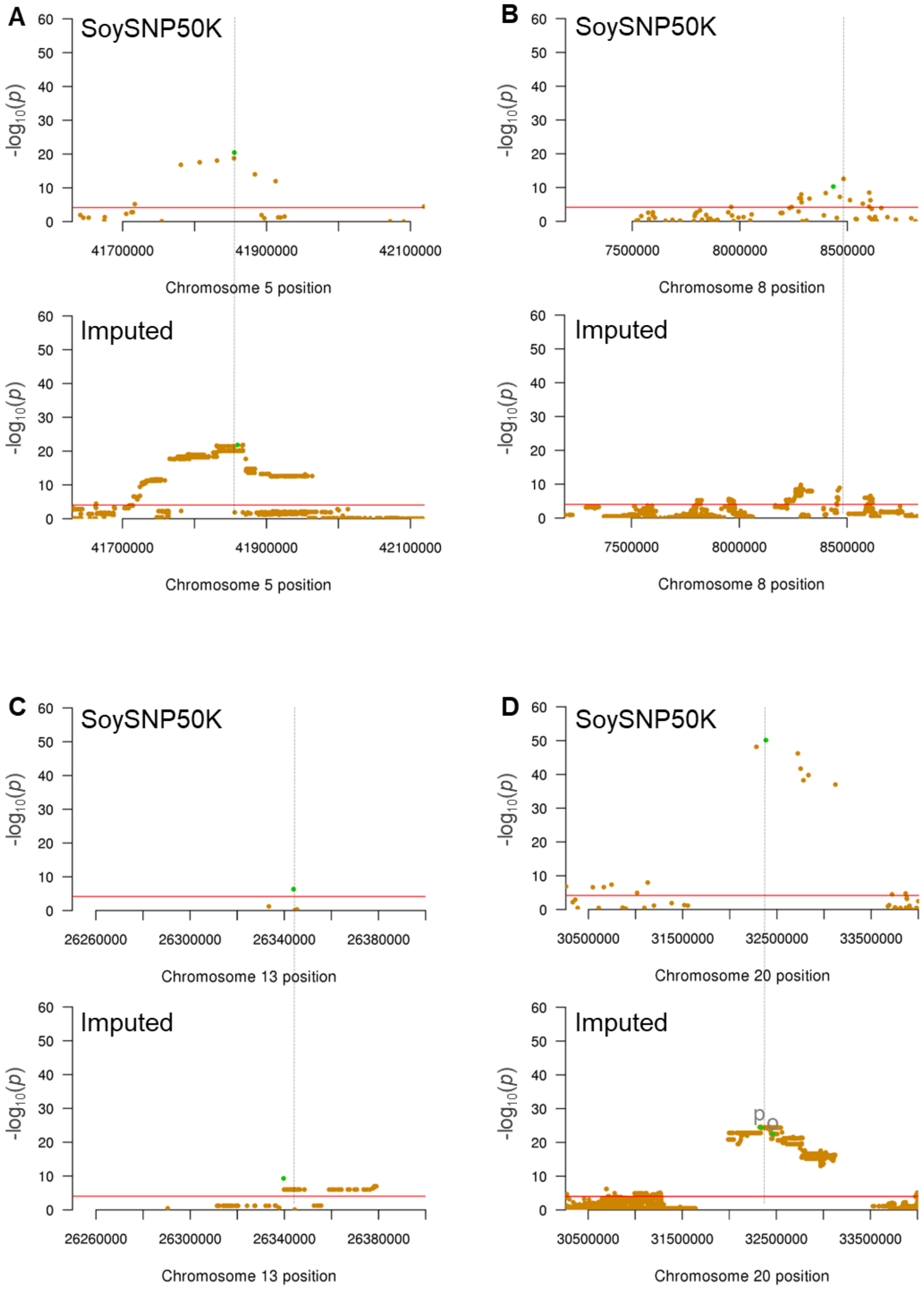

Supplementary Figure 19. Comparison of mvMLM-based GWAS results using unimputed (SoySNP50K) and imputed 3.1 million SNP data at major peaks on chromosomes. (**A**) A major peak on chromosome 5. The most significant SNP in the GWAS on unimputed data is indicated by dashed vertical lines for comparison. (**B**) A major peak on chromosome 8. (**C**) A major peak on chromosome 13. (**D**) A major peak on chromosome 20. A single peak SNP found in the MLMM analysis for each of peaks is in green and unique single peak SNPs for each of protein (p) and oil (o) on chromosome 20 were found in case of imputed data.

Supplementary Table 1. Whole-genome SNP, indel, structural variant distribution in 781 soybean genomes

Type		Population			
		Total	Glycine max	Glycine soja	Hybrid
Variants	SNPs	10,597,683	6,085,061	10,323,813	6,134,427
	Indels	1,414,161	826,168	1,371,041	764,400
	Structural variants	22,338	12,560	21,344	10,361
SNPs in genes	5' UTR	178,621	104,662	174,910	107,018
	CDS	397,869	230,796	386,815	239,617
	3' UTR	196,040	114,478	191,977	119,930
SIFT	Synonymous	170,427	99,806	166,930	105,539
	Nonsynonymous tolerant	149,077	89,916	145,017	92,679
	Nonsynonymous deleterious	70,795	36,992	67,613	37,268
	Stop-gain	6,266	3,274	5,991	3,308
	Stop-loss	656	435	638	442

Supplementary Table 2. Spearman rank's correlation coefficients between patterns of sequence variables (window size, 100 kb) observed across the genome. All variables were significantly correlated with each other at $P < 2.2e\text{-}16$

	Gene	SNP	Indel	Rho	GERP	LD distance	SV
gene		0.294	0.766	0.133	0.925	-0.786	0.361
SNP			0.692	0.297	0.314	-0.424	0.377
Indel				0.280	0.782	-0.811	0.485
Rho					0.109	-0.231	0.179
GERP						-0.800	0.358
LD							-0.389

SNP, single nucleotide polymorphism; indel, insertion/deletion; Rho, historical recombination rate; GERP, genomic evolutionary rate profiling; LD, linkage disequilibrium; SV, structural variant (> 50 base pairs).

Supplementary Table 3. Comparison of position numbers of SNPs significantly associated with either protein or oil on chromosome 20 between Glyma1 (Ws82.a1.v1) and Ws82.a2.v1

SNP (Ws82.a1.v1)	SNP (Ws82.a2.v1)		
Maker name with chromosome and position number	Marker name	Position on chromosome 20	comment
BARC_1.01_Gm_20_29594697_A_G	ss715637225	30,744,968	
BARC_1.01_Gm_20_29983050_A_G	ss715637240	31,129,249	
BARC_1.01_Gm_20_30930931_A_G	ss715637283	21,083,902	displaced
BARC_1.01_Gm_20_31150279_T_C	ss715637294	32,282,623	
BARC_1.01_Gm_20_31243150_C_T	ss715637299	32,384,035	
BARC_1.01_Gm_20_31436069_A_G	ss715637309	32,576,952	
BARC_1.01_Gm_20_31580769_A_G	ss715637315	32,721,955	
BARC_1.01_Gm_20_31610452_T_C	ss715637316	32,752,215	
BARC_1.01_Gm_20_31640038_A_G	ss715637318	32,781,800	
BARC_1.01_Gm_20_31687470_A_C	ss715637321	32,835,139	
BARC_1.01_Gm_20_31972955_G_A	ss715637329	33,121,197	

Supplementary References

1. Jeong, S. C. *et al.* Genetic diversity patterns and domestication origin of soybean. *Theor. Appl. Genet.* **132**, 1179–1193 (2019).
2. Schmutz, J. *et al.* Genome sequence of the palaeopolyploid soybean. *Nature* **463**, 178–183 (2010).
3. DePristo, M. A. *et al.* A framework for variation discovery and genotyping using next-generation DNA sequencing data. *Nat. Genet.* **43**, 491–498 (2011).
4. Carlson, J. B. & Lersten, N. R. Reproductive morphology. In *Soybeans: Improvement, production, and uses, 3rd edn* (Boerma, H. R. & Specht, J. E. eds.) 59–95 (ASA, CSSA, and SSSA, 2004).
5. Lu, B. R. Conserving biodiversity of soybean gene pool in the biotechnology era. *Plant Species Biol.* **19**, 115–125 (2004).
6. Ray, J. D., Kilen, T. C., Abel, C. A. & Paris, R. L. Soybean natural cross-pollination rates under field conditions. *Environ. Biosafety Res.* **2**, 133–138 (2003).
7. Fujita, R., Ohara, M., Okazaki, K. & Shimamoto, Y. The extent of natural cross-pollination in wild soybean (*Glycine soja*). *J. Hered.* **88**, 124–128 (1997).
8. Haun, W. J. *et al.* The composition and origins of genomic variation among individuals of the soybean reference cultivar Williams 82. *Plant Physiol.* **155**, 645–655 (2011).
9. Chia, J. M. *et al.* Maize HapMap2 identifies extant variation from a genome in flux. *Nat. Genet.* **44**, 803–807 (2012).
10. The 1000 Genomes Project Consortium. An integrated map of genetic variation from 1,092 human genomes. *Nature* **491**, 56–65 (2012).
11. Xu, X. *et al.* Resequencing 50 accessions of cultivated and wild rice yields markers for identifying agronomically important genes. *Nat. Biotechnol.* **30**, 105–111 (2012).
12. Menozzi, P., Piazza, A. & Cavalli-Sforza, L. of Human Gene Synthetic Frequencies Europeans. *Science* **201**, 786–791 (1978).
13. Raj, A., Stephens, M. & Pritchard, J. K. fastSTRUCTURE: variational inference of population structure in large SNP data sets. *Genetics* **197**, 573–589 (2014).
14. Lee, Y. G. *et al.* Development, validation and genetic analysis of a large soybean SNP genotyping array. *Plant J.* **81**, 625–636 (2015).
15. Dong, Z., Alexander, M. & Chuck, G. Understanding grass domestication through maize mutants. *Trends Genet.* **35**, 118–128 (2019).
16. Tian, Z. *et al.* Artificial selection for determinate growth habit in soybean. *Proc. Natl. Acad. Sci. U. S. A.* **107**, 8563–8568 (2010).
17. Dong, Y. *et al.* Pod shattering resistance associated with domestication is mediated by a NAC gene in soybean. *Nat. Commun.* **5**, 3352 (2014).
18. Sun, L. *et al. GmHs1-1*, encoding a calcineurin-like protein, controls hard-seededness in soybean. *Nat. Genet.* **47**, 939–943 (2015).
19. Zhang, D. *et al.* Elevation of soybean seed oil content through selection for seed coat shininess. *Nat. Plants* **4**, 30–35 (2018).
20. Swarm, S. A. *et al.* Genetic dissection of domestication-related traits in soybean through genotyping-by-sequencing of two interspecific mapping populations. *Theor. Appl. Genet.* **132**, 1195–1209 (2019).
21. Funatsuki, H. *et al.* Molecular basis of a shattering resistance boosting global dissemination of soybean. *Proc. Natl. Acad. Sci. U. S. A.* **111**, 17797–17802 (2014).
22. Simons, Y. B., Turchin, M. C., Pritchard, J. K. & Sella, G. The deleterious mutation load is insensitive to recent population history. *Nat. Genet.* **46**, 220–224 (2014).

23. Larson, G. *et al.* Current perspectives and the future of domestication studies. *Proc. Natl. Acad. Sci. U. S. A.* **111**, 6139–6146 (2014).
24. Bandillo, N. *et al.* A population structure and genome-wide association analysis on the USDA soybean germplasm collection. *Plant Genome* **8**, (2015).
25. Song, Q. *et al.* Fingerprinting soybean germplasm and its utility in genomic research. *G3 (Bethesda)* **5**, 1999–2006 (2015).
26. Anderson, C. A. *et al.* Data quality control in genetic case-control association studies. *Nat. Protoc.* **5**, 1564–1573 (2010).
27. Patil, G. *et al.* Dissecting genomic hotspots underlying seed protein, oil, and sucrose content in an interspecific mapping population of soybean using high-density linkage mapping. *Plant Biotechnol. J.* **16**, 1939–1953 (2018).
28. Burton, J. W. Quantitative genetics: Results relevant to soybean breeding. In *Soybeans: Improvement, production and uses, 2nd edn* (Wilcox, J. R. ed.) 211–247 (ASA, CSSA, and SSSA, 1987).
29. Lee, S. *et al.* Genome-wide association study of seed protein, oil and amino acid contents in soybean from maturity groups I to IV. *Theor. Appl. Genet.* **132**, 1639–1659 (2019).
30. Song, Q. *et al.* Development and evaluation of SoySNP50K, a high-density genotyping array for soybean. *PLoS One* **8**, e54985 (2013).
