# Supplementary material for: The patterns of deleterious mutations during the domestication of soybean
Source: Nat Commun. 2021 Jan 4;12:97. doi: 10.1038/s41467-020-20337-3 (PMC7782591; doi:10.1038/s41467-020-20337-3)
Supplement: Supplementary file 2 — Reporting Summary [file 41467_2020_20337_MOESM2_ESM.pdf]

nature research

Corresponding author(s): Soon-Chun jeong

Last updated by author(s): Nov 18, 2020

# Reporting Summary

Nature Research wishes to improve the reproducibility of the work that we publish. This form provides structure for consistency and transparency in reporting. For further information on Nature Research policies, see Authors & Referees and the Editorial Policy Checklist .

## Statistics

For all statistical analyses, confirm that the following items are present in the figure legend, table legend, main text, or Methods section.

| n/a | Confirmed | |
|---|---|---|
| ☐ | ☒ | The exact sample size (*n*) for each experimental group/condition, given as a discrete number and unit of measurement |
| ☐ | ☒ | A statement on whether measurements were taken from distinct samples or whether the same sample was measured repeatedly |
| ☐ | ☒ | The statistical test(s) used AND whether they are one- or two-sided<br>*Only common tests should be described solely by name; describe more complex techniques in the Methods section.* |
| ☒ | ☐ | A description of all covariates tested |
| ☐ | ☒ | A description of any assumptions or corrections, such as tests of normality and adjustment for multiple comparisons |
| ☐ | ☒ | A full description of the statistical parameters including central tendency (e.g. means) or other basic estimates (e.g. regression coefficient) AND variation (e.g. standard deviation) or associated estimates of uncertainty (e.g. confidence intervals) |
| ☐ | ☒ | For null hypothesis testing, the test statistic (e.g. *F*, *t*, *r*) with confidence intervals, effect sizes, degrees of freedom and *P* value noted<br>*Give P values as exact values whenever suitable.* |
| ☒ | ☐ | For Bayesian analysis, information on the choice of priors and Markov chain Monte Carlo settings |
| ☒ | ☐ | For hierarchical and complex designs, identification of the appropriate level for tests and full reporting of outcomes |
| ☐ | ☒ | Estimates of effect sizes (e.g. Cohen's *d*, Pearson's *r*), indicating how they were calculated |

*Our web collection on statistics for biologists contains articles on many of the points above.*

## Software and code

Policy information about availability of computer code

| | |
|---|---|
| Data collection | No software was used. |
| Data analysis | GATK v4.0.1.2, FastQC v0.11.3, BWA v0.1.12, Picard tools v1.134, VCFtools v0.1.15, Eigensoft v7.2, FastStructure v1.0, DISTRUCT v1.1, FastEPRR v1.0, Circos v0.69-6, PopLDdecay v3.31, GCTA v1.92.1, XP-CLR v1.0, Beagle v5.0, SIFT 4G, GERP++, PLINK v1.9, GEMMA v0.98.1, MLMM for R, LASTZ/MULTIZ pipeline (LASTz v1.04.00 and MULTIZ v012109) at http://genomewiki.ucsc.edu/index.php/DoBlastzChainNet.pl, ete3 v3.1.1, est-sfs v2.03. SAMtools v1.9, BCFtools v1.9, Seqtk v1.2, PSMC v0.6.5-r67, qqman v0.1.4 in R package, and phyloFit v1.5. We described the usage of each software with options in the METHODS. |

For manuscripts utilizing custom algorithms or software that are central to the research but not yet described in published literature, software must be made available to editors/reviewers. We strongly encourage code deposition in a community repository (e.g. GitHub). See the Nature Research guidelines for submitting code & software for further information.

## Data

Policy information about availability of data

All manuscripts must include a data availability statement. This statement should provide the following information, where applicable:

- Accession codes, unique identifiers, or web links for publicly available datasets
- A list of figures that have associated raw data
- A description of any restrictions on data availability

Although 16 of the original data (NCBI SRA accession numbers ERX2248648-ERX48662 and ERR953473) have been released in conjunction with prior publications, we uploaded raw reads in fastq format for all 855 final accessions to NCBI SRA with SRA accession number PRJNA555366. Large datasets including SNPs, indels, SV calls, SIFT scores, GERP scores, and ancestral state of CDS SNP variants and the source data for Supplementary Fig. 6 are available from figshare repository (https://figshare.com/projects/Soybean_haplotype_map_project/76110). Data supporting the findings of this work are available within the paper and its Supplementary Information files. A reporting summary for this Article is available as a Supplementary Information file. The datasets and plant materials generated and analyzed during the current study are available from the corresponding author upon request. The source data underlying Figure 4a-c as well as Supplementary Figures 2, 6, 11, and 13 are provided as a Source Data file. Known variant sites for soybean, uniref90, and annotation of G. max Wm82.a2.v1 were downloaded from NCBI dbSNP

Build 144 (https://www.ncbi.nlm.nih.gov/projects/SNP/snp_summary.cgi?build_id=144), UniProt (https://www.uniprot.org/), and EnsemblPlants (ftp://ftp.ensemblgenomes.org/pub/plants/release-44/gff3/glycine_max), respectively. Plant reference genome sequences were downloaded from RefSeq database (https://www.ncbi.nlm.nih.gov/refseq/) and URGI database (https://urgi.versailles.inra.fr/Species/Vitis). The phylogenetic tree was downloaded from Dryad database (https://datadryad.org/resource/doi:10.5061/dryad.63q27.2).

# Field-specific reporting

Please select the one below that is the best fit for your research. If you are not sure, read the appropriate sections before making your selection.

- [x] Life sciences
- [ ] Behavioural & social sciences
- [ ] Ecological, evolutionary & environmental sciences

For a reference copy of the document with all sections, see nature.com/documents/nr-reporting-summary-flat.pdf

# Life sciences study design

All studies must disclose on these points even when the disclosure is negative.

| | |
|---|---|
| Sample size | Non-redundant 781 accessions as a haplotype map panel consisted of 418 Glycine max (domesticated soybean) including 332 landraces and 86 improved lines, 345 Glycine soja (wild soybean), and 18 hybrid (G. max x G. soja) accessions. The G. soja and hybrid accessions were obtained from China, Korea, Japan, and the Russian Far East that cover the worldwide distribution of soybean. The 18 hybrid accessions were sufficient because they were used only for analyses of population structure and diversity patterns. Several hundreds of G. max and G. soja accessions provided sufficient powers for genome-wide comparisons between G. max and G. soja and the G. soja sample size is the largest used up to date. |
| Data exclusions | Of the 855 samples, 74 that unexpectedly showed higher than two thirds of heterozygous to homozygous non-reference SNPs ratios or inbreeding coefficient per individual of less than 0.8 were excluded from the haplotype map panel. The high heterozygosity was not expected because domesticated and wild soybean is predominantly selfing and the sequenced lines had gone through at least two generations of single-seed descent for the current study. Thus, this was done to remove the possibility of confounding by cross-pollination in soybean |
| Replication | We replicated genome resequencing of 17 soybeans accessions to test whether their high heterozygosity rates were due to experimental errors or the natural conditions of plants. The high heterozygous rate did not change except for three trials. As the 781 sample set as a haplotype map panel did not contain any redundant accession, subsequent genomic analyses were not affected by the replication experiments. |
| Randomization | Not applicable due to this study being comparisons of data sets between domesticated and wild soybean groups and being correlation analyses between individual variants and phenotypes for genome-wide association studies. |
| Blinding | Not applicable due to this study being compared all accessions to each other. |

# Reporting for specific materials, systems and methods

We require information from authors about some types of materials, experimental systems and methods used in many studies. Here, indicate whether each material, system or method listed is relevant to your study. If you are not sure if a list item applies to your research, read the appropriate section before selecting a response.

## Materials & experimental systems

| n/a | Involved in the study |
|---|---|
| [x] | [ ] Antibodies |
| [x] | [ ] Eukaryotic cell lines |
| [x] | [ ] Palaeontology |
| [x] | [ ] Animals and other organisms |
| [x] | [ ] Human research participants |
| [x] | [ ] Clinical data |

## Methods

| n/a | Involved in the study |
|---|---|
| [x] | [ ] ChIP-seq |
| [x] | [ ] Flow cytometry |
| [x] | [ ] MRI-based neuroimaging |
