# Supplementary material for: The patterns of deleterious mutations during the domestication of soybean
Source: Nat Commun. 2021 Jan 4;12:97. doi: 10.1038/s41467-020-20337-3 (PMC7782591; doi:10.1038/s41467-020-20337-3)
Supplement: Supplementary file 3 — Description of Additional Supplementary Files [file 41467_2020_20337_MOESM3_ESM.pdf]

Description of Supplementary Data Files

File Name: Supplementary Data 1
Description: List of soybean accessions sequences.

File Name: Supplementary Data 2
Description: Allele frequencies, SIFT prediction, and GERP score, and ancestral or derived status of reference alleles of nonsynonymous SNPs identified at CDS regions of genes that have been cloned with implication of domestication selection in soybean.

File Name: Supplementary Data 3
Description: List of genes that contain novel or standing domestication alleles. Allele frequencies, SIFT prediction, and GERP score, and ancestral or derived status of reference alleles of nonsynonymous SNPs located at candidate domestication genes whose nonsynonymous alleles have been almost fixed in the domesticated population (allele frequency > 0.99) and rare in the wild population (allele frequency < 0.01) screened genome-wide.
